# Evidence-domain translation in the Lyme disease controversy over persistent post-treatment symptoms

**DOI:** 10.3389/fcimb.2026.1913455

**Published:** 2026-09-07

**Authors:** Teo Susnjak, Lorenz Brehme, Kunal Garg, Gordana Avramovic, Leona Gilbert

**Affiliations:** 1 School of Mathematical and Computational Sciences, College of Sciences, Massey University, Auckland, New Zealand; 2 University of Innsbruck, Department of Computer Science, Innsbruck, Austria; 3 Tezted Oy, Jyväskylä, Finland; 4 Department of Infectious Diseases, Mater Misericordiae University Hospital, Dublin, Ireland; 5 School of Medicine, University College Dublin, Dublin, Ireland

**Keywords:** chronic lyme disease, computational discourse analysis, epistemic filters, evidence translation, large language models, lyme disease, post-treatment lyme disease syndrome, scientific controversy

## Abstract

The debate between post-treatment Lyme disease syndrome (PTLDS) and chronic Lyme disease (CLD) reflects different views about the causes and treatment of persistent symptoms attributed to Lyme disease, including symptoms that continue after recommended antibiotic treatment. This study examines how competing positions in the controversy draw on different scientific evidence domains, and how findings from those domains are extended into broader clinical and mechanistic claims. We analysed a 2000–2024 literature corpus using a prompt-optimised three-model large language model ensemble to classify abstracts by stance, theme and model-derived study design. We then conducted targeted full-text audits of selected retreatment and treatment-duration trials, and preclinical persistence studies. Across the higher-volume evidence domains, model-derived claim orientations differed by study-design tier. Observational studies and commentaries showed PTLDS-oriented distributions, whereas case reports or series, animal models, and *in vitro* studies showed CLD-oriented distributions. The smaller RCT and guideline tiers were also PTLDS-oriented, but the small numbers and wide confidence intervals made this direction uncertain. The clinical trial audit showed that some trials reported short-term or symptom-specific improvements, while the interpretation of these findings depended on their durability, endpoint consistency, eligibility criteria, treatment burden, and safety. The citation-conditioned preclinical audit identified findings that support several candidate mechanisms and generate hypotheses for further study, but these findings did not by themselves establish viable infection as the cause of persistent human symptoms or demonstrate the efficacy of prolonged antimicrobial treatment. The findings show a recurring distinction between mechanism-level evidence and the evidence needed to establish patient-level causation or durable clinical benefit. They map how these different forms of evidence are distributed and translated across the PTLDS–CLD literature.

## 1 Introduction

Lyme borreliosis is a tick-borne infection whose recognised acute manifestations generally respond to recommended antimicrobial therapy, although treatment response and recovery vary by manifestation and disease stage (Lantos et al., 2021). A clinically important subset of treated patients nevertheless reports persistent fatigue, pain, cognitive symptoms, or functional impairment. In a prospective cohort of 1,084 patients included in the primary analysis, persistent symptoms were more frequent after treated Lyme borreliosis than in population and tick-bite comparison cohorts, although the background prevalence in both comparison cohorts was substantial (Ursinus et al., 2021). In research and clinical guidance, posttreatment Lyme disease syndrome (PTLDS) generally denotes persistent symptoms after documented and appropriately treated Lyme disease, subject to temporal, diagnostic, and exclusion criteria (Rebman and Aucott, 2020; Lantos et al., 2021; Marques, 2022). “Chronic Lyme disease” (CLD) has been used more heterogeneously for persistent symptoms attributed to ongoing or incompletely treated infection, unrecognised infection, co-infection, or a broader chronic multisystem illness (Feder et al., 2007; Cameron et al., 2014; Marques, 2022). These usages carry different diagnostic thresholds, causal assumptions, evidentiary standards, and assessments of the benefits and harms of antimicrobial retreatment. Neither category encompasses every patient with persistent symptoms after Lyme disease. A broader descriptive classification has therefore been proposed for symptoms attributed to Lyme disease that fall outside strict PTLDS criteria, while retaining uncertainty about causation (Baarsma and Hovius, 2024).

The relevant evidence domains answer different questions. RCTs estimate average treatment effects in defined populations; observational studies characterise associations and clinical heterogeneity; animal and *in vitro* studies test candidate mechanisms within defined experimental systems; and qualitative research examines patient experience, diagnostic uncertainty and institutional trust. Retreatment trials have not shown a consistent pattern of durable net benefit for the regimens and diagnostically bounded populations studied, although individual trials differed in eligibility, interventions, endpoints, and secondary signals (Klempner et al., 2001; Krupp et al., 2003; Fallon et al., 2008; Berende et al., 2016; Dersch et al., 2024). Proposed explanations for persistent symptoms include viable microbial persistence in a subset of patients, retained microbial antigens, inflammatory or autoimmune dysregulation, altered neurological processing or central sensitisation, and unrelated or overlapping conditions. Their relative contributions remain uncertain and may differ among patient subgroups (Batheja et al., 2013; Rebman and Aucott, 2020; Baarsma and Hovius, 2024). In this study, mechanistic plausibility denotes support within defined experimental systems for one or more such pathways; it does not establish which pathway causes persistent symptoms in patients. Preclinical, immunological, xenodiagnostic, and biomarker findings can support investigation of these hypotheses, but do not by themselves establish the cause of persistent human symptoms or the efficacy of prolonged treatment. The dispute therefore concerns both the findings and the rules used to translate evidence across domains.

Our previous study mapped publication patterns, stance distributions, and recurring themes in 8,630 abstracts published from 2000 through 2024 (Susnjak et al., 2026). The present study asks a different question: how model-derived claim orientations are distributed across study designs and how selected clinical and preclinical findings are extended into mechanistic, therapeutic, and guideline-level claims. Its new components are prompt-optimised ensemble reclassification, study-design and evidence-domain mapping, descriptive stance-orientation odds and targeted full-text evidence-translation audits. The analysis therefore distinguishes what each evidence domain can directly support from the broader mechanistic, therapeutic, or guideline-level claims sometimes drawn from it.

Large-scale evidence mapping requires consistent classification across a broad literature (Haddaway et al., 2020). LLMs can assist literature classification and extraction, but their outputs depend on the taxonomy, prompt wording, model endpoint and evaluation procedure (Gilardi et al., 2023; Dennstädt et al., 2024; Adam et al., 2025). We therefore treated prompt instructions as auditable decision rules, optimised them against inherited expert-agreed examples, applied a provider-diverse three-model ensemble for increased reliability, retained the model outputs and adjudication records and paired abstract-level mapping with targeted full-text appraisal. The resulting labels provide a structured representation of published claim orientation, enabling comparison across evidence domains and targeted examination of how findings are translated into broader mechanistic, therapeutic and clinical arguments.

## 2 Materials and methods

### 2.1 Study aim and design

This descriptive evidence-domain and evidence-translation study linked abstract-level stance labels to model-derived study-design categories and targeted full-text appraisal. It examined distributions of published claims rather than the correctness of individual PTLDS- or CLD-aligned interpretations. It did not rank the quality of every publication, estimate causal effects, or infer treatment efficacy from publication counts. We conducted a retrospective secondary analysis of a previously screened English-language abstract corpus, followed by stance, theme, and study-design labelling, evidence-domain mapping and targeted full-text analysis of selected high-leverage domains.

### 2.2 Corpus provenance and relationship to the prior study

**Figure 1:**
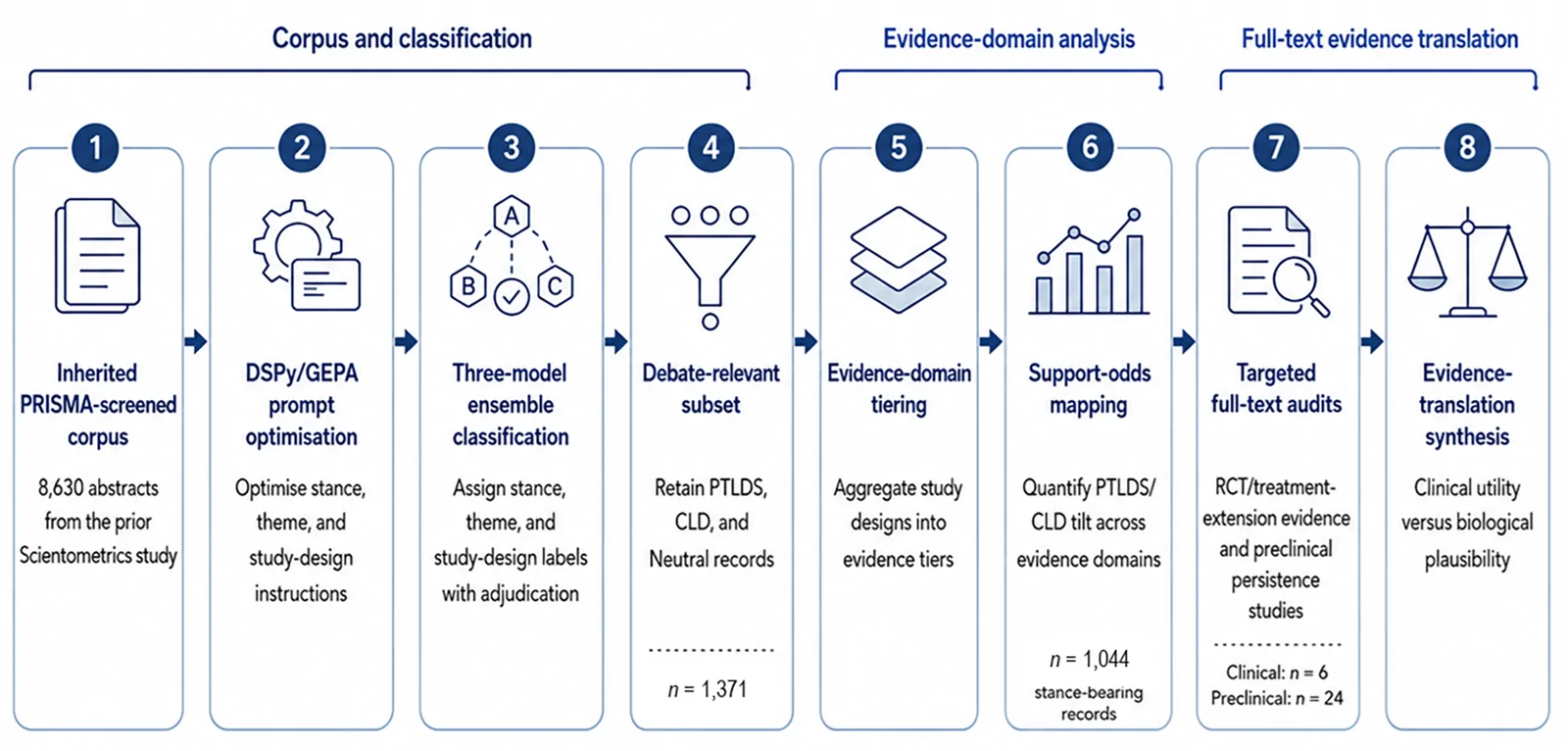
Methodological workflow of the follow-up evidence-translation study. The figure summarises the pipeline from the inherited PRISMA-screened corpus through DSPy/GEPA LLM prompt optimisation, three-model ensemble classification, evidence-domain tiering, stance-orientation odds mapping, targeted full-text audits, and final evidence-translation synthesis. The final integrated analysis contained 1,371 debate-relevant records, of which 1,044 carried a directional stance label.

This study builds directly on the screened corpus and classification framework developed in our prior scientometric mapping of the Lyme disease controversy (Susnjak et al., 2026). The prior study assembled a 25-year corpus of Lyme-related scholarly records using PRISMA-style screening and prespecified inclusion and exclusion criteria. That work established the bibliographic and abstract-level sampling frame, the five-class stance architecture, an expert-labelled reference set, and an eight-theme taxonomy capturing the main recurring points of contention in the PTLDS–CLD literature. The present study reused the prior sampling frame and coding architecture but generated the evidence-domain analysis reported here. **Table 1** separates inherited and new components. **Figure 1** summarises the workflow.

**Table 1:** Components inherited from the prior study and components introduced in the present study.

Component	Prior study	Present study
Screened 2000–2024 corpus and bibliographic frame	Created	Reused unchanged
Five-class stance architecture and eight-theme taxonomy	Developed	Reused for reclassification
Two-expert 150-record exercise and 91 exactagreement subset	Created	Reused for prompt development
DSPy/GEPA prompt optimisation and three-provider production ensemble	Not performed	Introduced
Study-design labels, evidence-domain tiers, and stance-orientation mapping	Not performed	Introduced
Targeted RCT and preclinical full-text audits	Not performed	Introduced

### 2.3 Corpus and eligibility

The analysis used the fixed PRISMA-filtered Lyme disease abstract corpus from the prior study, covering publications from 1 January 2000 to 31 December 2024. Eligible records contained English-language bibliographic and abstract information and had passed the prior relevance screening. Publications appearing after this period were used only to contextualise the methods or interpretation and did not contribute to corpus classification, evidence-domain estimates, citation-conditioned sampling, or the full-text audits. Given the large size of the corpus, abstract-level classification was used because the aim was to map large-scale claim orientation across the published literature, rather than conducting a full-text systematic review of every included record. Abstracts are the most consistently available structured representation of article-level claims across bibliographic databases; however, it is also recognised that they omit methodological caveats and full evidentiary context. For this reason, abstract-level mapping was paired with targeted full-text appraisal of high-leverage clinical and preclinical domains in this study.

### 2.4 Inherited reference material and theme taxonomy

Three resources from the prior study were reused. First, the screened corpus and bibliographic metadata provided the sampling frame for reclassification. Second, the prior study provided human reference material for stance/routing prompt development. In that earlier study, two domain experts independently classified a random sample of 150 abstracts using the operational labels Supports PTLDS, Supports CLD, Neutral, Unrelated, and Animal Study. They assigned the same label to 91 abstracts and different labels to 59 abstracts. Human–human agreement across the complete sample was moderate (Cohen’s *κ* = 0.501), reflecting the interpretive difficulty of the task. We used the 91 exact-agreement labels as fixed prompt-development material, divided into training, internal-validation, and held-out prompt-development partitions. The 59 disputed records were excluded from optimisation because they lacked an adjudicated reference label. The prior study reported agreement of its classifier with the 91 human-agreed labels (*κ* = 0.709; accuracy = 0.769; macro-F1 = 0.754), with the weakest performance for Neutral. These metrics describe the classifier used in the prior study rather than the GEPA-optimised production ensemble used here. The inherited theme taxonomy also underwent targeted expert review in the prior study: 50 abstract–justification pairs, comprising 100 theme assignments, were reviewed, with 96% of assignments confirmed. These resources supported reference-based prompt development but did not independently validate all model-derived stance, theme, and study-design labels in the present analysis. Third, we reused the eight-theme taxonomy developed and validated in the prior study comprising Active Infection vs. Post-Infectious Immune Activity; Diagnostic Complexity and Uncertainty; Therapeutic Controversies and Antibiotic Efficacy; Neurocognitive and Neuropsychiatric Manifestations; Immune Dysregulation and Autoimmune Mechanisms; Patient-Centred Experiences and Advocacy; Mechanisms of Pathogen Persistence and Biofilm Formation; and Sociocultural and Ethical Factors. This taxonomy was treated as fixed, while theme assignments were re-estimated using new DSPy/GEPA-revised prompts and a three-model ensemble.

### 2.5 DSPy/GEPA prompt optimisation

Prompt optimisation refined the written classification instructions without altering model weights or the predefined label categories. GEPA applied candidate instructions to the inherited training examples, examined errors against their fixed labels, and proposed revised instructions in natural language. Candidate wording was selected using a separate internal-validation partition, while the held-out partition was reserved from prompt selection. This procedure addresses the sensitivity of biomedical screening performance to instruction wording, output structure, and task framing (Dennstädt et al., 2024; Cao et al., 2025). The 91 inherited exact-agreement records were divided into 54 training, 18 internal-validation, and 19 held-out prompt-development records. This compact development set was used to optimise and select instructions, consistent with the data-efficient setting for which DSPy prompt optimisers were developed (Khattab et al., 2023; Opsahl-Ong et al., 2024; Agrawal et al., 2025); it was not intended to estimate corpus-wide classification performance. Because it contained only exact-agreement cases, it may overrepresent clearer examples and underrepresent ambiguous boundary cases.

For each labelling task, the prompt instruction was treated as an optimisable decision protocol. Candidate instructions were generated using the training partition and selected using the internal-validation partition. The held-out partition was used to assess the selected instructions during prompt development. Candidate model configurations were compared during development, and the final production ensemble comprised gpt-5-mini, Grok-4 Fast, and Gemini 2.0 Flash. Full implementation details, including candidate models, optimiser roles, the feedback metric, optimisation budget, and recorded generation settings, are provided in the **Supplementary Methods**.

### 2.6 Classification pipeline

The present study applied three abstract-level labelling stages followed by evidence-domain mapping and targeted full-text appraisal. First, each abstract was assigned one mutually exclusive stance or routing label: Supports PTLDS, Supports CLD, Neutral, Unrelated, or Animal Study. Under the operational definitions, Supports PTLDS represented an overall post-infectious or non-persistent framing, or a conclusion against additional prolonged antimicrobial treatment, whereas Supports CLD represented an overall ongoing-infection or persistence framing, or a conclusion favouring extended antimicrobial treatment. Neutral indicated no clear directional position. Animal Study was used as a routing category rather than as a clinical stance. It identified non-human studies that did not make direct claims about human PTLDS or CLD, while allowing animal studies with explicit human-relevant interpretive claims to be assigned to a substantive stance category. For evidence-domain analyses, animal-model studies could enter the debate-relevant set only if the abstract was classified as Supports PTLDS, Supports CLD, or Neutral based on explicit interpretive claims relevant to the controversy. Thus, “Animal Study” as a routing label and “animal model” as an evidence-domain tier were analytically distinct.

Second, debate-relevant abstracts, defined as Supports PTLDS, Supports CLD, or Neutral, were assigned theme labels using the inherited eight-theme taxonomy. Third, each debate-relevant abstract was assigned one primary study-design label and, where applicable, secondary study-design labels. These labels were then aggregated into evidence-domain tiers, including RCTs, controlled non-randomised studies, observational studies, case reports or series, animal models, *in vitro* studies, diagnostic accuracy studies, methods or assay-development studies, reviews or meta-analyses, guidelines, editorials, commentaries, imaging-only studies, and other non-clinical contributions.

### 2.7 Three-model ensemble and adjudication

Each abstract-classification task was performed independently by three provider-diverse LLM endpoints selected on development-set performance, provider diversity, availability, structured-output suitability, and operational feasibility: gpt-5-mini, Grok-4 Fast, and Gemini 2.0 Flash. This design reduced reliance on one model family. A subsequent screening study similarly found distinct sensitivity and specificity profiles across LLMs and reported that combining outputs increased sensitivity while also increasing false-positive inclusion (Oami et al., 2025). The taxonomy and operational criteria were fixed before production classification. Each model received its own optimised prompt without access to the other models’ outputs, and a two-of-three majority determined the model-derived label where two models agreed. Complete three-way stance/routing disagreements entered a separate adjudication invocation using x-ai/grok-4-fast through OpenRouter. The invocation received the abstract, three candidate labels, and their short rationales and applied the same prespecified definitions. Because this step reused one production endpoint, it resolved the final label without adding an independent adjudicator. Complete three-way disagreements in primary study design were retained as unresolved design records without an additional model call.

Pre-resolution stability was summarised using the proportions of unanimous, two-to-one, and complete three-way disagreement outcomes, together with pairwise agreement and multi-rater agreement statistics. These measures characterise consistency among the production endpoints but do not establish correctness against a human reference standard. Full results are reported in **Supplementary Table 1**.

### 2.8 Thematic and study-design labelling

For debate-relevant abstracts, defined as records classified as Supports PTLDS, Supports CLD, or Neutral, we applied two additional labelling stages. First, we assigned thematic labels using the eight-theme taxonomy inherited from the prior study. Because the taxonomy had already been generated through multi-model thematic extraction and expert consolidation, the present study treated it as an established coding framework. The follow-up analysis assessed its adequacy for the reclassified debate-relevant subset and then applied it consistently under the DSPy/GEPA-assisted ensemble pipeline.

To assess whether the inherited taxonomy remained adequate for the reclassified debate-relevant corpus, each model reviewed batches of abstracts for recurring themes not represented in the eight-theme framework. No additional recurring theme was retained. For final thematic assignment, each model generated candidate labels for each abstract, and retained labels were determined under a strict cross-model consensus rule to limit inflation of theme prevalence and prioritise discriminative assignment over maximal coverage.

Second, each debate-relevant abstract was assigned one primary study-design label and, where applicable, up to three secondary study-design labels from a predefined study-design taxonomy. Primary study-design labels were then aggregated into evidence-domain tiers for analysis. These tiers included RCTs, controlled non-randomised studies, observational studies, case reports or series, animal models, *in vitro* studies, diagnostic accuracy studies, methods or assay-development studies, reviews or meta-analyses, guidelines, editorials, commentaries, imaging-only studies, and other non-clinical contributions. The primary study-design labels described document design as reported in titles and abstracts; they did not assess study quality, risk of bias, or evidentiary validity. Study-design instructions were refined on a development subset for which all three models assigned the same primary category. Because this subset was model-derived rather than human-adjudicated, the resulting study-design and evidence-domain labels were used for descriptive aggregation and selection of domains for full-text audit.

### 2.9 Analytical denominators

**Table 2** summarises the main analytical denominators used in the follow-up study. The operational starting point was the abstract corpus that entered the prior semantic classification workflow. Larger pre-classification screening denominators reported in the prior study are treated as preceding corpus-construction stages rather than as direct denominators for the present evidence-domain analysis. Study-design agreement was calculated on 1,373 pre-resolution debate-relevant records. Reconciliation with the final integrated dataset produced a net reduction of two records, leaving 1,371 debate-relevant records, of which 1,044 carried directional stance labels. Agreement statistics therefore use *n* = 1,373, whereas the final evidence-domain and stance analyses use *n* = 1,371. Record-level reconciliation details are provided in the **Supplementary Methods**.

**Table 2:** Analytical denominators linking the prior corpus to the follow-up evidence-domain analysis.

Stage	*n*	Basis
Prior operational abstract corpus used for semantic classification	8,630	Corpus inherited from the prior Scientometrics study and used as the starting frame for this follow-up analysis
Pre-resolution study-design inputs	1,373	Workflow-stage debate-relevant records classified independently by the three study design models; denominator used for intermodel agreement
Debate-relevant abstracts	1,371	Records classified as Supports PTLDS, Supports CLD, or Neutral in the final integrated dataset
Stance-bearing abstracts	1,044	Records classified as Supports PTLDS or Supports CLD in the final integrated dataset; Neutral records excluded
Evidence-domain labelled debate-relevant records	1,371	Debate-relevant records assigned a primary study-design/evidence-domain label in the final integrated dataset
RCT-labelled stance-bearing abstract records used in stance orientation odds mapping	20	Abstract records classified as RCTs and Supports PTLDS or Supports CLD; used in **Figure 3**
Unique RCT or controlled treatment publications identified for full-text audit	13	Deduplicated or relevance-screened treatment records retained as full-text audit candidates
Retreatment or treatment-extension trials discussed in **Table 5**	6	Four direct persistent-symptom retreatment or longer-term therapy trials and two adjacent treatment-duration trials
Preclinical full-text extraction set	24	Citation-conditioned, design-balanced sample of animal or *in vivo* and *in vitro* studies

The 8,630 abstracts constitute the operational starting set for this secondary analysis; preceding screening counts are reported in the prior study. The 1,373-record denominator applies to pre-resolution study-design agreement, whereas final evidence-domain analyses use the reconciled 1,371-record dataset.

RCT counts differ because stance-orientation analysis uses abstract-level records, while the full-text audit uses deduplicated and relevance-screened publications. Of 20 RCT-labelled stance-bearing abstracts and 13 retained treatment candidates, four direct persistent-symptom trials and two adjacent treatment-duration trials were selected for detailed appraisal.

### 2.10 Evidence-domain stance-orientation mapping

To characterise how stance-bearing claims were distributed across evidence domains, we estimated stance-orientation odds within each evidence-domain tier. Within each tier, stance-orientation odds were calculated as the number of Supports PTLDS records divided by the number of Supports CLD records. These are within-tier odds rather than odds ratios relative to a reference tier. Neutral abstracts were excluded because they did not express a directional stance. Tier-specific odds greater than one indicated a PTLDS tilt, while odds below one indicated a CLD tilt. Two-sided 95% confidence intervals were calculated as Wald intervals on the log odds, using variance 1*/a* + 1*/b*, where *a* and *b* are the Supports PTLDS and Supports CLD counts. The analysis code applied a 0.5 Haldane correction only when either cell was zero; no displayed all-theme tier required this correction. Results were plotted on a log scale to aid comparison across tiers with different counts. This analysis was used descriptively rather than as a causal test. Its purpose was to identify which evidence domains were disproportionately associated with each stance and to guide targeted full-text appraisal of high-leverage domains where cross-tier translation appeared most consequential.

### 2.11 Targeted full-text evidence-translation audit

The full-text phase was designed as an evidence-translation audit rather than as an exhaustive systematic review or meta-analysis. The RCT audit focused on retreatment and treatment-extension trials that are frequently mobilised in guideline reasoning about persistent symptoms after Lyme disease treatment. From 13 retained clinical candidates, we selected four trials directly evaluating retreatment or longer-term therapy in persistent-symptom populations (Klempner, Krupp, Fallon, and Berende) and two adjacent randomised treatment-duration trials in disseminated or late Lyme disease (Oksi and Dattwyler). Protocols, commentaries, secondary or biomarker reports, coinfection-specific interventions, and acute erythemamigrans duration studies were not included in the detailed appraisal. For each clinical trial, we extracted the enrolled population, diagnostic or serological eligibility criteria, treatment comparison, primary or major secondary treatment signal, durability interpretation, safety considerations, and the defensible evidentiary conclusion supported by the trial. For retreatment and treatment-extension trials, we also recorded trial design, sample size, primary endpoint, major secondary signals, follow-up duration, adverse events, and applicability to the PTLDS–CLD treatment question so that trial-internal findings could be distinguished from external applicability to broader contested CLD populations. Additional structured extraction fields are provided in the **Supplementary Material**.

The preclinical audit focused on a bounded, citation-conditioned, design-balanced subset of animal or *in vivo* and *in vitro* studies relevant to the persistent infection versus post-infectious interpretation. Selection was guided by evidence-domain tier, stance imbalance in the abstract-level stance-orientation analysis, citation uptake in guideline and review literature, and the need to cover major translation mechanisms. For each preclinical paper, we extracted the model system, treatment context, persistence measure, viability claim, directly supported inference, and broader clinical interpretation sometimes attached to the finding. This design allowed us to examine how evidence moves from mechanism to clinical argument without treating the selected full-text set as statistically representative of all Lyme disease research.

### 2.12 Selection of the preclinical extraction subset

The preclinical full-text phase was designed as a targeted evidence-translation audit rather than as an exhaustive review of all animal and *in vitro* Lyme studies. We therefore selected a bounded, purposive sample of 24 preclinical papers comprising 12 animal or other *in vivo* studies and 12 *in vitro* studies, using a citation-conditioned procedure intended to identify studies that were both mechanistically relevant and demonstrably influential in the guideline and review literature. The sampling frame was first restricted to corpus-derived records classified as active-infection preclinical studies, comprising animal-model and *in vitro* papers relevant to the persistence versus post-infectious interpretation. We then identified 53 eligible active-infection guideline, review, and meta-analysis source documents, including neutral evidence synthesis papers, because the purpose of this step was to map evidence flow rather than to reproduce stance-odds denominators. Reference lists from the available full-text conversions were extracted and matched against the preclinical target frame using DOI-exact matches as high-confidence evidence and title/year overlap as evidence requiring further review.

The final 24-paper subset was constructed to balance citation influence with design diversity and inferential coverage. Citation overlap supplied the initial influence signal, while inclusion also required representation across the main evidence types and translation mechanisms relevant to the persistence versus post-infectious interpretation. Specifically, the subset was constructed to include (i) animal or *in vivo* persistence models and *in vitro* mechanistic studies in equal numbers; (ii) papers cited by at least one guideline or review record where possible; (iii) studies addressing the major translation mechanisms identified in the abstract-level analysis, including persistence after antibiotic exposure, antigen or DNA detection versus viability, immune-evasion or immune-dysregulation mechanisms, pleomorphic or drug tolerant forms, and diagnostic caution around nucleic-acid detection; and (iv) papers that represented both infection-persistence arguments and post-infectious or immune-mediated counter-interpretations. This procedure treated the 24 papers as a citation-conditioned, design-balanced extraction set rather than as a statistically representative sample of all preclinical Lyme research.

Before finalising the subset, we verified the eligibility of all source documents and manually reviewed citation matches not supported by an exact DOI. The 53 eligible guideline, review, and meta-analysis documents yielded 4,304 reference strings and 147 citation links to 70 unique preclinical studies. From these, 24 papers were selected using the prespecified design and mechanism criteria. This procedure retained a transparent connection among corpus eligibility, citation uptake, reference matching, and design– mechanism coverage. The 24-paper subset is therefore an influential, citation-conditioned evidence-flow sample rather than a prevalence estimate for all preclinical Lyme research. Detailed matching diagnostics, source verification, unmatched candidates, and sensitivity records are provided in the **Supplementary Material**.

### 2.13 Transparency and data handling

The corpus-level inputs comprised bibliographic records and abstracts obtained through the prior literature screening workflow, while the targeted evidence-translation audits used published full-text articles. No private patient-level or unpublished clinical data were analysed. We retained the classification inputs, compiled prompts, operational definitions, endpoint identifiers, available generation settings, aggregation and disagreement-resolution rules, original model outputs, derived labels, and analysis scripts. Available workflow metadata place the runs in November 2025, record temperatures of 0 or 1 depending on the task, establish that no seed parameter was supplied to model calls, and identify JSON as the structured-output format. These materials support reconstruction of the reported analysis, but future runs may differ because hosted providers can change model weights, routing, system instructions, safety layers, or serving infrastructure while retaining a public endpoint name. Detailed implementation information and the limits of the available run metadata are reported in the **Supplementary Methods**.

## 3 Results

### 3.1 Overarching patterns, trends and themes

**Table 3** reports final model-derived stance labels under the prespecified ensemble and adjudication procedure. The present study re-estimated rather than carried forward the earlier labels, and some records moved between Neutral, directional and routing categories. These distributions provide a structured representation of abstract-level claim orientation and form the basis for the subsequent comparisons across themes and evidence domains.

**Table 3:** Distribution of model-derived stance labels among debate-relevant abstracts.

Stance	Number of records	Percentage (%)
Neutral	327	24
Supports CLD	509	37
Supports PTLDS	535	39

**Table 4** shows that model-derived stance labels were not uniformly distributed across themes in this corpus. Theme counts are tagged occurrences rather than mutually exclusive study sets because an abstract may carry several themes. The largest theme, Active Infection vs. Post-Infectious Immune Activity (*n* = 844), was moderately tilted towards Supports CLD (43.5%) relative to Supports PTLDS (38.6%), with a smaller Neutral proportion (17.9%). Abstracts assigned to this theme were therefore more frequently given a directional stance than a Neutral label. The subsequent evidence-domain analysis examined which study designs were most frequently represented among infection-centred and post-infectious claims.

**Table 4:** Distribution of model-derived stance labels among theme-tagged abstract occurrences.

Theme	n	Neutral (%)	PTLDS (%)	CLD (%)
Active Infection vs. Post-Infectious Immune Activity	844	17.9	38.6	43.5
Diagnostic Complexity and Uncertainty	792	30.6	39.1	30.3
Therapeutic Controversies and Antibiotic Efficacy	522	17.4	45.2	37.4
Neurocognitive and Neuropsychiatric Manifestations	237	30.0	38.8	31.2
Immune Dysregulation and Autoimmune Mechanisms	233	16.3	53.6	30.0
Patient-Centred Experiences and Advocacy	120	50.0	31.7	18.3
Mechanisms of Pathogen Persistence and Biofilm Formation	121	3.3	2.5	94.2
Sociocultural and Ethical Factors	70	51.4	30.0	18.6

Stance and theme were assigned in separate abstract-level classification tasks. Percentages represent the model-derived stance labels among abstracts carrying each theme, rather than a stance inherent to the theme itself. For patient-centred and sociocultural records, the stance label reflects the abstract’s overall causal, diagnostic, or therapeutic framing rather than necessarily reflecting the patient’s own interpretation of illness.

Diagnostic Complexity and Uncertainty (*n* = 792) had a larger Neutral proportion (30.6%) and less separation between Supports PTLDS (39.1%) and Supports CLD (30.3%) than the Active Infection theme. This distribution indicates greater abstract-level neutrality within diagnostically oriented records and motivated examination of the evidence domains represented in this theme. Therapeutic Controversies and Antibiotic Efficacy (*n* = 522) was more frequently classified as Supports PTLDS (45.2%) than Supports CLD (37.4%). This pattern is consistent with a greater prevalence of PTLDS-aligned risk–benefit and controlled-efficacy framing among abstracts assigned to the therapeutic theme.

Immune Dysregulation and Autoimmune Mechanisms (n=233) is the most PTLDS-skewed theme (53.6%), suggesting that when immune-mediated explanations are foregrounded, authors more often frame persistent symptoms as post-infectious sequelae rather than ongoing infection. In contrast, Mechanisms of Pathogen Persistence and Biofilm Formation showed the strongest CLD orientation in the model-coded theme assignments, with 94.2% of theme-tagged occurrences classified as Supports CLD. This indicates that, within this corpus and coding framework, persistence-oriented mechanistic language was usually positioned as part of a CLD-aligned argument rather than as a neutral mechanistic observation. The predominantly neutral composition of Patient-Centred Experiences and Advocacy (50.0%) and Sociocultural and Ethical Factors (51.4%) suggests that these themes often function as meta-level commentary on lived experience, contested authority, and clinical relationships rather than as direct mechanistic argumentation. This pattern motivated the subsequent stratification of theme-linked claims by study-design tier and publication type.

**Figure 2:**
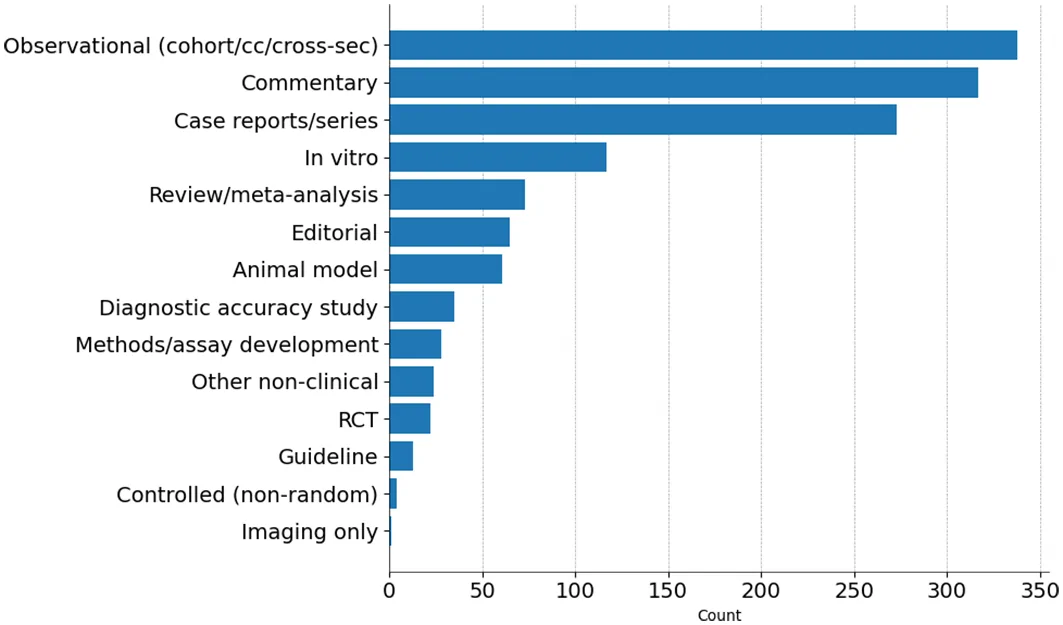
Distribution of model-derived primary study-design labels among the 1,371 debate-relevant abstracts in the final integrated analysis, using the specified taxonomy and three-model ensemble procedure. In the figure, “cc” denotes case–control and “cross-sec” denotes cross-sectional.

**Figure 2** shows that the model-derived debate-relevant set contained many observational studies and non-empirical discourse formats, with fewer RCTs and controlled non-randomised studies. The broad domains support different inferences: observational and case-based designs describe associations and heterogeneity; preclinical studies test plausibility and mechanisms; and RCTs estimate average treatment effects under defined conditions. Publication volume therefore should not be equated with evidentiary strength.

### 3.2 Stance-orientation odds across evidence domains

**Figure 3:**
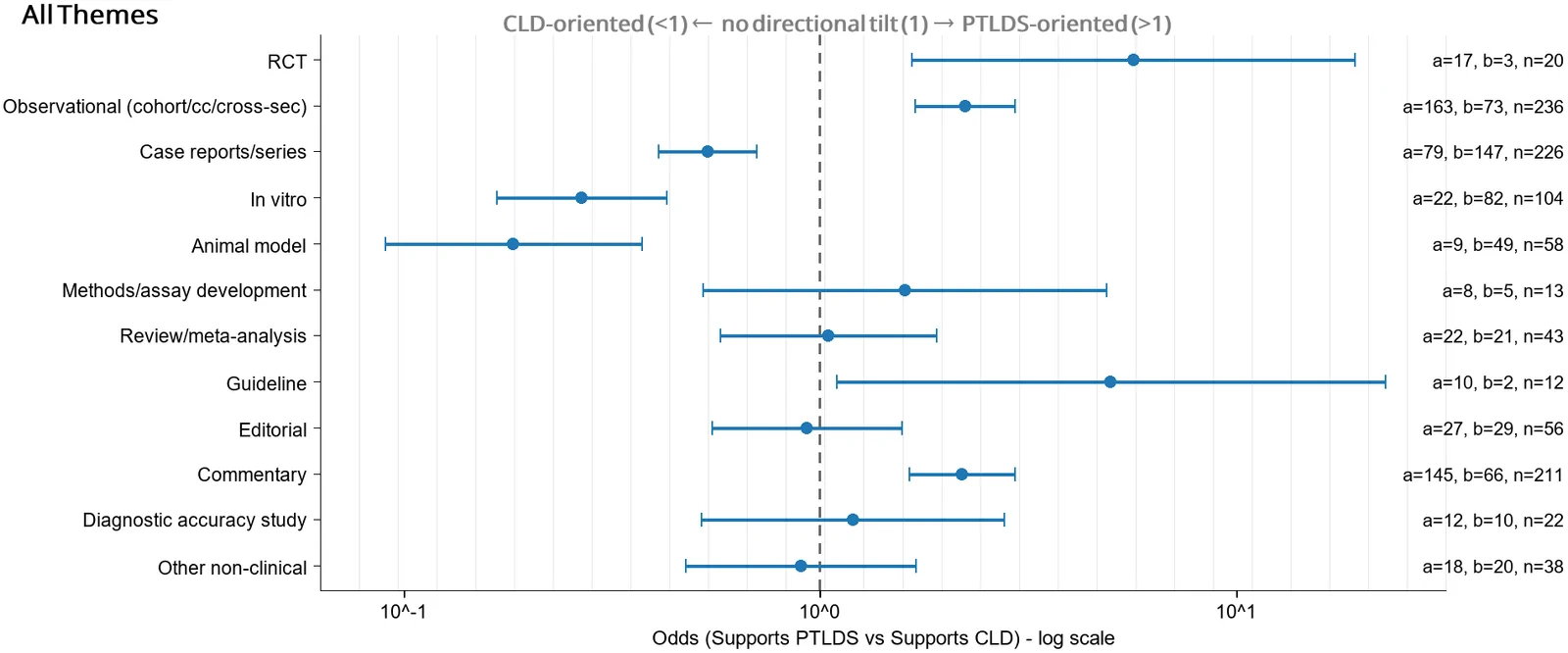
Stance-orientation odds across evidence-domain tiers for all stance-bearing abstracts. Values represent the odds that an abstract-level claim was classified as Supports PTLDS rather than Supports CLD within each evidence-domain tier. Neutral records were excluded. Values greater than 1 indicate PTLDS orientation; values below 1 indicate CLD orientation. In the annotations, *a* is the number classified as Supports PTLDS, *b* is the number classified as Supports CLD, and *n* = *a*+*b* is the number of stance-bearing abstracts in that tier. Odds are plotted on a log scale with confidence intervals. The figure displays 1,039 of the 1,044 stance-bearing records in the final dataset. Five records in sparse tiers were omitted: four controlled non-randomised records (two Supports PTLDS and two Supports CLD) and one imaging-only record (Supports PTLDS).

**Figure 3** reports stance-orientation odds among the 1,044 model-derived stance-bearing abstracts. High-volume tiers supplied the more stable descriptive patterns: observational studies were PTLDS-oriented (*a* = 163, *b* = 73), case reports/series were CLD-oriented (*a* = 79, *b* = 147), commentaries were PTLDS-oriented (*a* = 145, *b* = 66), and animal-model and *in vitro* tiers were CLD-oriented (*a* = 9, *b* = 49 and *a* = 22, *b* = 82, respectively). The RCT group contained 20 abstracts (*a* = 17, *b* = 3), and the guideline group contained 12 (*a* = 10, *b* = 2). Both groups were PTLDS-oriented, but their small sizes and wide confidence intervals make the apparent direction uncertain; we therefore use these results to guide closer full-text appraisal rather than as precise summaries of the wider RCT or guideline literature.

Before aggregation and tie resolution, the three production models agreed unanimously on 5,926 of 8,630 stance/routing records (68.7%), reached a two-to-one majority on 2,560 (29.7%), and produced complete three-way disagreement on 144 (1.7%). The 144 stance/routing disagreements entered model based adjudication. For primary study design, the models agreed unanimously on 1,011 of the 1,373 workflow-stage inputs (73.6%), reached a two-to-one majority on 325 (23.7%), and produced complete three-way disagreement on 37 (2.7%). The 37 study-design disagreements were retained as unresolved design records rather than model-adjudicated. These figures describe inter-model consistency before resolution; they do not establish correctness. **Supplementary Table 1** provides the corresponding pairwise agreement estimates and Fleiss’ *κ* statistics and distinguishes this workflow-stage denominator from the final 1,371-record analytic file.

#### 3.2.1 Stance-orientation odds within the core etiological theme

**Figure 4:**
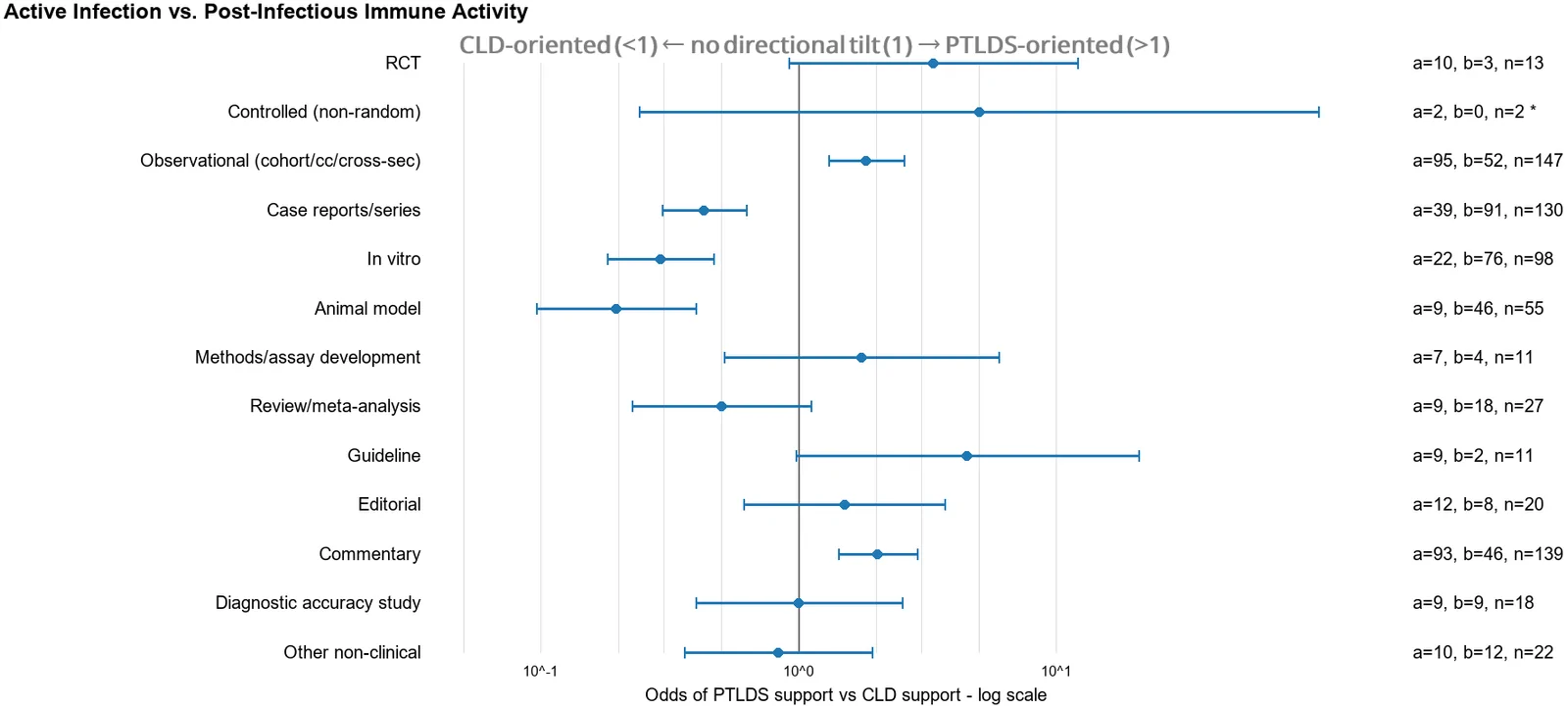
Stance-orientation odds across evidence-domain tiers restricted to stance-bearing abstracts tagged with the *Active Infection vs. Post-Infectious Immune Activity* theme. Values represent the odds that an abstract-level claim was classified as Supports PTLDS rather than Supports CLD within each evidence-domain tier. Neutral records were excluded. Values greater than 1 indicate PTLDS orientation; values below 1 indicate CLD orientation. In the annotations, *a* is the number classified as Supports PTLDS, *b* is the number classified as Supports CLD, and *n* = *a* + *b* is the number of stance-bearing abstracts in that tier. An asterisk indicates that the prespecified 0.5 zero-cell correction was applied.

**Figure 4** repeats the analysis for stance-bearing abstracts tagged with *Active Infection vs. Post-Infectious Immune Activity*. RCTs (*a* = 10, *b* = 3, odds ≈ 3.33), guidelines (*a* = 9, *b* = 2, odds ≈ 4.50), observational studies (*a* = 95, *b* = 52, odds ≈1.83), and commentaries (*a* = 93, *b* = 46, odds ≈2.02) were PTLDS-oriented. Animal models (*a* = 9, *b* = 46, odds ≈0.20), *in vitro* studies (*a* = 22, *b* = 76, odds ≈ 0.29), and case reports/series (*a* = 39, *b* = 91, odds ≈ 0.43) were CLD-oriented. Diagnostic accuracy studies were balanced (*a* = 9, *b* = 9, odds =1.00). Reviews/meta-analyses had a CLD-leaning point estimate (*a* = 9, *b* = 18, odds =0.50), but the interval crossed unity. The controlled non-randomised tier contained two Supports PTLDS records and no Supports CLD records; the plotted estimate therefore used the prespecified 0.5 zero-cell correction and remained highly imprecise. These active-theme results support directional interpretation rather than precise effect-size claims, particularly in sparse tiers.

These figures map the distribution of model-derived claim orientations across evidence domains. The patterns indicate that different forms of evidence were associated with different orientations within the corpus and provide a basis for identifying domains that warranted closer examination. The targeted full-text audits complement this corpus-level mapping by examining how selected clinical and preclinical findings were interpreted and extended into broader mechanistic and therapeutic claims. Thus, the two analytical levels connect large-scale evidence-domain patterns with more detailed appraisal of high-leverage studies.

### 3.3 Mechanisms of translation in the RCT evidence tier

RCTs provide the most direct evidence on patient-level treatment benefit, durability, and safety under defined eligibility criteria. The abstract-level analysis identified 20 stance-bearing records labelled as RCTs. After deduplication and relevance screening for treatment-extension or retreatment relevance, 13 unique RCT or controlled treatment publications were retained as audit candidates. The resulting six-trial appraisal comprised four trials in persistent-symptom populations and two adjacent duration trials in disseminated or late Lyme disease. This purposive set covered the clinical contexts most often mobilised in the controversy; it was not an exhaustive systematic review and does not represent the complete Lyme RCT literature.

The key translation issue is how trial signals are interpreted when symptom-specific or short-term benefits are weighed against endpoint consistency, durability, diagnostic specificity, treatment burden, and safety. The RCT appraisal therefore identifies more than a simple absence of signal. However, transient or domain-specific responses do not by themselves establish persistent infection or justify extended antibiotic treatment.

#### 3.3.1 Treatment signal, endpoint consistency, and durability

Several trials reported findings that acquire different interpretive force depending on which evidentiary criterion is prioritised. Fallon et al. (2008) reported short-term cognitive improvement after intravenous ceftriaxone in a narrowly defined group with post-treatment Lyme encephalopathy, but the cognitive benefit was not sustained after antibiotic discontinuation. Krupp et al. (2003) reported significant improvement in severe fatigue after intravenous ceftriaxone, without corresponding improvement in cognitive function or in the experimental CSF OspA marker. These findings show that the RCT evidence includes symptom-related treatment signals in selected populations.

The clinical interpretation of those signals depends on durability, breadth, endpoint consistency, and treatment burden. In the audited guideline-oriented sources, recommendations placed substantial weight on sustained patient-level benefit, consistency across clinically meaningful outcomes, adverse events, and the burden of intravenous or prolonged antimicrobial therapy (Lantos et al., 2021; Dersch et al., 2024). Selected CLD-aligned guidance and reviews assigned greater interpretive weight to symptom-specific or short-term responses, persistence hypotheses, possible responder subgroups, and the possibility that tested regimens did not exhaust the relevant treatment space (Cameron et al., 2014; Adkison and Embers, 2023). Published reassessments likewise differed in the weight assigned to fatigue-specific and short-term signals, durability, safety, and generalisability (DeLong et al., 2012; Klempner et al., 2013). The disagreement therefore concerns how far a trial signal can support inference from symptom response to mechanism and from mechanism to treatment recommendation.

#### 3.3.2 Safety, treatment burden, and risk–benefit translation

Safety considerations remain central to the clinical interpretation of retreatment trials, particularly when benefits are transient, symptom-specific, not durable, or not clearly superior to shorter treatment. This is especially relevant where interventions require intravenous access, prolonged antimicrobial exposure, or regimens with non-trivial adverse-event risks.

**Table 5** summarises this risk–benefit translation across the selected direct and adjacent trial evidence. The table separates eligibility criteria, treatment comparison, treatment signal, durability, safety considerations, and the evidentiary conclusion supported by each trial. Klempner et al. (2001); Krupp et al. (2003); Fallon et al. (2008) speak most directly to retreatment in documented post-treatment populations, although with different eligibility criteria and endpoints. Berende et al. (2016) addresses longer-term oral antibiotic therapy after initial ceftriaxone in patients with persistent symptoms attributed to Lyme disease. Oksi et al. (2007); Dattwyler et al. (2005) contribute adjacent treatment-duration evidence in disseminated or late Lyme disease. Additional extraction detail for the clinical trial audit is provided in the **Supplementary Material**.

**Table 5:** Diagnostic eligibility, treatment signal, durability, and safety considerations in selected direct and adjacent Lyme treatment trials.

Study	Eligibility/population	Treatment comparison and main signal	Durability interpretation	Safety considerations	Defensible evidence-translation conclusion
Klempner et al. (2001)	Two parallel trials: seropositive patients and seronegative patients with well-documented, previously treated Lyme disease and persistent symptoms.	Intravenous ceftriaxone for 30 days followed by oral doxycycline for 60 days versus matched placebo. No significant improvement over placebo on SF-36 physical or mental health-component outcomes at day 180.	No durable clinical advantage was demonstrated at the main 180-day endpoint.	Adverse events occurred in both antibiotic and placebo groups; serious treatment-related events were reported in antibiotic-treated patients.	The study includes both seropositive and clinically documented seronegative patients, so its evidentiary scope is best understood as documented post- treatment disease rather than symptoms attributed to CLD without comparable documentation.
Krupp et al. (2003)	Post-Lyme syndrome with severe fatigue at least six months after antibiotic therapy, with physician-documented erythema migrans or CDC-defined late Lyme disease confirmed by positive ELISA and Western blot serology.	Intravenous ceftriaxone for 28 days versus intravenous placebo. Fatigue improved significantly with ceftriaxone, but cognition and CSF OspA did not.	Outcome was assessed at six months; the authors interpreted the fatigue-only response cautiously because it was not accompanied by cognitive or laboratory improvement.	Treatment-associated adverse events requiring hospitalisation occurred in the trial, including events in both study arms; interpretation requires attention to event type and treatment attribution in the full report.	Demonstrates a statistically significant fatigue-specific treatment signal in a documented post-Lyme syndrome population, but does not demonstrate cognitive benefit, microbiological clearance, or a generalisable rationale for routine additional intravenous ceftriaxone. The finding is best interpreted as hypothesis-generating for symptom-defined subgroups rather than as evidence for broad retreatment efficacy.
Fallon et al. (2008)	Post-treatment Lyme encephalopathy with well-documented prior Lyme disease, at least three weeks of prior intravenous antibiotic therapy, current positive IgG Western blot, and objective memory impairment.	Intravenous ceftriaxone for 10 weeks versus intravenous placebo. Cognitive performance improved at week 12 across cognitive domains, with secondary improvement in some physical symptoms.	Cognitive benefit was not sustained to week 24 after antibiotics were discontinued, although some secondary pain and physical-functioning improvements persisted.	Adverse events related to medication or PICC line occurred more often in ceftriaxone-treated patients, with no permanent injury reported.	Supports a short-term cognitive treatment signal in a narrowly defined post-treatment Lyme encephalopathy subgroup, but does not establish a durable cognitive treatment strategy or prove that persistent viable infection was the mechanism of response.
Oksi et al. (2007)	Disseminated Lyme borreliosis, classified as definite or possible using symptoms, signs, and laboratory results. Adjacent treatmentduration evidence in disseminated Lyme borreliosis.	Patients were randomised to oral amoxicillin or placebo for 100 days, with both groups first receiving intravenous ceftriaxone for 21 days.	Clinical outcome was judged over 6–12 months; the authors concluded that adjunct oral antibiotics were not justified after initial intravenous ceftriaxone.	No serious antibiotic adverse effects occurred. Diarrhoea was common, and rare C. difficile events were reported.	Best interpreted as evidence against extended adjunct oral therapy after initial intravenous ceftriaxone in disseminated Lyme borreliosis, with relevance to evidence-translation questions about extended therapy, but limited direct applicability to PTLDS– CLD retreatment claims.
Dattwyler et al. (2005)	Late Lyme disease with objective neurologic, rheumatologic, or dermatologic manifestations and serological reactivity to B. burgdorferi. Randomisation occurred before serological results, but seronegative patients were excluded from the efficacy analysis.	Ceftriaxone for 14 days versus 28 days. Clinical cure rates at last evaluation were similar between groups, although there were more treatment failures in the 14-day group.	Follow-up suggested continued improvement after treatment; the study was not powered to identify subgroups that might benefit from longer treatment.	Treatment discontinuation due to adverse events was significantly more frequent in the 28-day group.	Provides adjacent evidence on late-Lyme treatment duration and illustrates how serological eligibility restricts the population to which the findings apply.
Berende et al. (2016)	Persistent symptoms attributed to Lyme disease, either temporally related to proven Lyme disease or accompanied by positive IgG or IgM immunoblot evidence.	All groups received open-label intravenous ceftriaxone for two weeks, then 12 weeks of doxycycline, clarithromycin– hydroxychloroquine, or placebo. Longerterm-therapy did not improve SF-36 physicalcomponent scores more than placebo.	No additional longer-term treatment effect was found at later follow-up visits.	Adverse events were common, but rates were similar among groups; serious drug-related adverse events occurred during the open-label ceftriaxone phase, with no serious drug-related adverse event during the randomised phase.	Evidence weighs against longer-term oral antibiotic therapy after initial ceftriaxone in patients with persistent symptoms attributed to Lyme disease under the study’s entry criteria.

“Treatment signal” refers to statistically significant or clinically emphasised findings on primary or major secondary outcomes as reported in the trial papers. The studies differ in eligibility criteria, treatment structure, outcome targets, and relationship to the PTLDS–CLD retreatment question. Klempner, Krupp, and Fallon are retreatment trials in documented post-treatment populations, although with different entry criteria and outcome targets. Berende is a persistent-symptom trial comparing longer-term oral regimens after open-label ceftriaxone. Oksi and Dattwyler contribute adjacent treatment-duration evidence in disseminated or late Lyme disease and are interpreted here in relation to evidence translation rather than as direct PTLDS–CLD retreatment trials.

Across the audited trials, extended or repeated antibiotic regimens did not produce a consistent pattern of durable net clinical benefit in the diagnostically bounded populations studied. Their clinical interpretation therefore depends on the durability and breadth of any treatment signal, considered alongside treatment burden and safety. Evidence of a treatment effect and the strength of a clinical recommendation remain separate judgements because recommendation development also considers certainty, harms, burden, feasibility, and applicability (Guyatt et al., 2008). In the four trials directly involving persistent-symptom populations, active treatment courses ranged from 28 days to 14 weeks; the adjacent Oksi trial tested a 100-day oral extension after 21 days of ceftriaxone. The audited trials therefore do not address indefinite or substantially longer courses, and this evidentiary gap does not establish efficacy or safety for untested durations. These findings constrain conclusions about the regimens and populations examined, while questions concerning viable persistence or treatment-responsive subgroups require evidence designed to address those propositions directly.

#### 3.3.3 Eligibility, diagnostic specificity, and external validity

A further translation issue concerns how trial eligibility shapes the population to which RCT findings can be generalised. Internal validity concerns the treatment contrast within the defined trial conditions, whereas external validity concerns applicability beyond the eligibility, intervention, comparator, outcome, and follow-up conditions (Rothwell, 2005). The pivotal retreatment and treatment-extension trials generally required documented prior Lyme disease, defined clinical manifestations, serological evidence, or a temporally specified relationship between persistent symptoms and a recognised Lyme disease episode. These criteria preserve diagnostic specificity and reduce heterogeneity, but they also narrow the population to which trial findings can be directly applied.

This distinction is especially important for interpreting Klempner et al. (2001), one of the central retreatment trials in the PTLDS–CLD debate. The study reported two parallel placebo-controlled trials: one enrolling seropositive patients and one enrolling seronegative patients, with both groups required to have well-documented, previously treated Lyme disease and persistent post-treatment symptoms. Its findings therefore apply to documented post-treatment populations, including a clinically documented seronegative group, rather than to the broader population of patients whose symptoms are attributed to chronic Lyme disease without comparable documentation.

Across the wider RCT set, eligibility criteria varied in clinically important ways. Krupp et al. (2003) enrolled patients with post-Lyme syndrome and severe fatigue at least six months after antibiotic therapy, but required physician-documented erythema migrans or a CDC-defined late manifestation of Lyme disease confirmed by positive ELISA and Western blot serology. Fallon et al. (2008) used a narrower neurological entry frame, requiring well-documented prior Lyme disease, at least three weeks of prior intravenous antibiotic therapy, current positive IgG Western blot, and objective memory impairment. Berende et al. (2016) used a broader persistent-symptom attribution framework, enrolling patients whose symptoms were temporally related to proven Lyme disease or accompanied by positive IgG or IgM immunoblot evidence. Oksi et al. (2007) contributes adjacent treatment-duration evidence in disseminated Lyme borreliosis, with patients randomised to adjunct oral amoxicillin or placebo while both groups received an initial 21-day course of intravenous ceftriaxone.

Dattwyler et al. (2005) provides a further adjacent example of how diagnostic specificity shapes the analysed trial population. It was an open-label randomised comparison of 14 versus 28 days of ceftriaxone for late Lyme disease with objective neurologic, rheumatologic, or dermatologic manifestations. Randomisation occurred before serological results were available, but patients with negative serology were subsequently excluded from the efficacy analysis. In total, 58 of 201 randomised patients were excluded for failure to meet serological criteria (Dattwyler et al., 2005). This illustrates how documentation requirements can materially restrict the evidence base in adjacent late-Lyme treatment trials.

The central evidence-translation issue is the trade-off between diagnostic specificity and external validity. From a trialist or guideline perspective, restrictive eligibility criteria make the average treatment effect more interpretable by reducing the risk that persistent, non-specific symptoms from unrelated causes will dilute the trial estimate. From a CLD-aligned or patient-advocacy perspective, the same restrictions may limit applicability to patients whose attribution to Lyme disease is clinically contested because documentation is incomplete, atypical, or disputed. **Table 5** summarises this issue across the pivotal and adjacent evidence by separating eligibility criteria, treatment signal, durability, safety considerations, and the evidentiary conclusion supported by each trial.

### 3.4 Mechanisms of translation in the preclinical evidence tier

The treatment audit illustrates how selected clinical trial findings are translated into guideline-oriented risk–benefit reasoning. To examine the distinct mechanistic evidence stream, we conducted a targeted audit of 24 preclinical papers, comprising 12 animal or other *in vivo* studies and 12 *in vitro* studies. This citation-conditioned, design-balanced subset was assembled to trace influential evidence flows and compare the inferential boundaries of findings generated across different preclinical systems, rather than to estimate the prevalence of persistence findings across the wider literature.

The main result is that the preclinical tier provides experimental-system-specific support for several candidate mechanisms more strongly than it supports direct clinical treatment claims. The inferential reach of individual findings varied according to the experimental system and outcome measured. Animal post-antibiotic findings, retained antigenic material, immune-evasion mechanisms, drug-tolerant phenotypes and tissue pathology supported biological mechanisms within the systems studied, although they differed in what they established about organism viability. Nucleic-acid detection, altered morphology and *in vitro* antibiotic tolerance supported more limited inferences about viable persistence, human symptom causation and clinical treatment efficacy. Across the 24 papers, 14 involved a post-antibiotic or drug-exposure context, eight did not, one examined retained *B. burgdorferi* peptidoglycan in human Lyme arthritis samples, and one had an unclear treatment context. The mechanisms represented in the set included animal post-antibiotic persistence, non-human-primate tissue/pathology findings, antigen or peptidoglycan persistence, immune evasion, immune dysregulation, matrix-metalloproteinase-mediated tissue pathology, cellular invasion, complement resistance, drug-tolerant persister cells, round-body or cystic morphology, and diagnostic caution around nucleic-acid detection. The selected studies thus encompass several distinct pathways through which persistent organisms, retained microbial material and host responses have been linked to hypotheses concerning ongoing symptoms.

#### 3.4.1 Detection of microbial material versus evidence of viability

A central evidentiary distinction concerns what constitutes evidence of infection after treatment. In the audited set, persistence was operationalised through culture or culture reactivation, PCR or other nucleic-acid detection, antigen or peptidoglycan detection, microscopy, xenodiagnostic or tissue signals, immune-response readouts, and drug-tolerance assays. These measurements carry different inferential weight. Human xenodiagnosis illustrates this distinction. In a first-in-human feasibility study, most treated participants had negative results, and detection of *B. burgdorferi* DNA associated with one participant did not establish viable organisms (Marques et al., 2014). Within the audited preclinical set, culture or reactivation findings provided more direct evidence relevant to viability than isolated nucleic-acid, antigen, or morphology findings, while remaining specific to the experimental system and not establishing human symptom causation. DNA-positive/culture-negative findings, antigen persistence, and morphology-based observations require more cautious interpretation because they can be compatible with either viable persistence or non-viable material. Interpretation also depended on the experimental model and measurement used. Non-human-primate pathology was suggestive rather than clinically decisive; DNA persistence in ceftriaxone-treated mice did not establish viability; matrix-metalloproteinase induction indicated host pathology rather than microbial persistence; and anti-TNF-associated reactivation represented an immunomodulation model rather than a direct rationale for antimicrobial retreatment.

#### 3.4.2 Antibiotic-tolerance evidence and trial-protocol interpretation

The *in vitro* subset shows how antibiotic-tolerance findings generate hypotheses about alternative regimens, drug combinations, pharmacodynamic conditions, or outcome measures that may merit clinical testing. These findings do not establish that the regimens used in existing clinical trials were inadequate, nor do they demonstrate that alternative or prolonged regimens would be safe or effective in PTLDS or CLD populations. Their warranted inferential role is trial generation rather than direct clinical prescription.

#### 3.4.3 Cross-tier translation and inferential reach

Across the selected full-text material, two recurrent cross-tier translation patterns were identified. The first used mechanistic possibility—including residual organisms, protected niches, immune evasion, persister forms, or morphology-dependent tolerance—to question whether existing RCTs fully cover the relevant biological mechanisms, treatment regimens, or patient subgroups. The second used patient-level evidence on efficacy, durability, treatment burden and safety to delimit what can be recommended clinically, even where persistent-infection mechanisms remained plausible within experimental systems. The audited material therefore indicates that the disagreement concerns not only whether preclinical persistence signals exist, but also how far those signals can be extended from animal and *in vitro* models to human symptoms, from detection to viability and from biological possibility to treatment recommendations. Accordingly, preclinical findings are complementary to, rather than a substitute for, patient-level treatment evidence. Animal persistence, antigen persistence, immune-evasion mechanisms, and *in vitro* drug-tolerance phenotypes can support mechanistic hypotheses and may inform biomarker development and targeted clinical trial design. Without additional clinical evidence, these findings do not establish that viable infection causes persistent symptoms in populations described as PTLDS or CLD, or that prolonged or non-standard antimicrobial regimens provide durable net clinical benefit.

## 4 Discussion

The descriptive analysis identified different distributions of abstract-level claim orientation across model-derived evidence domains. Among the better-populated tiers, observational studies and commentaries were PTLDS-oriented, whereas case reports or series and the animal-model and *in vitro* tiers were CLD-oriented. These corpus-level patterns were complemented by targeted full-text audits examining how selected clinical and preclinical findings were extended into broader mechanistic and treatment claims. Within the audited material, PTLDS-aligned interpretations foregrounded durable patient-level benefit, endpoint consistency, diagnostic eligibility, treatment burden, and safety, whereas CLD-aligned interpretations foregrounded persistence mechanisms, retained microbial material, protected niches, drug tolerance, and the possibility of treatment-responsive subgroups. Together, these analyses show how findings generated within different evidence domains acquired different significance when translated into broader clinical and mechanistic arguments.

The observed distribution is consistent with different evidence tiers serving distinct inferential roles within the controversy. RCTs provide direct evidence on patient-level clinical utility, durability, and safety under defined eligibility criteria. Preclinical studies provide support within defined experimental systems for candidate mechanisms and generate hypotheses requiring further clinical investigation. Research that connects these tiers more directly will require measures capable of distinguishing viable persistence, retained microbial material, and post-infectious host responses; clinically coherent patient classifications that do not presuppose one mechanism; and trials that test prospectively defined mechanistic hypotheses using clinically meaningful outcomes (Baarsma and Hovius, 2024). Human immune studies provide preliminary evidence of biological heterogeneity: post-treatment CCL19 concentrations were associated with later outcome groups in a prospective cohort, and a recent cohort identified differences in circulating Tcell phenotypes associated with PTLD status and symptom patterns (Aucott et al., 2016; Girgis et al., 2025). These associative findings do not establish a single causal mechanism or a validated treatment-responsive subgroup. Studies published after the corpus window provide further context for the distinction between viable persistence and retained microbial material: a prospective xenodiagnostic cohort found little evidence of detectable organisms after treatment while acknowledging limited sensitivity for very low-level or inaccessible persistence, and a murine study demonstrated tissue persistence of *B. burgdorferi* peptidoglycan and associated systemic responses without establishing viable organisms (Marques et al., 2026; McClune et al., 2025). The inferential boundaries summarised in **Table 6** reflect established distinctions among association, causal inference, external validity, certainty of evidence, and recommendation strength (Hill, 1965; Rothwell, 2005; Guyatt et al., 2008).

**Table 6:** Principal evidence-domain claims and defensible inferential boundaries in the PTLDS–CLD controversy.

Evidence domain	What it can support	What it cannot support alone	Illustrative overextension
RCTs	Average treatment effects, safety, and durability under defined eligibility criteria	Effects in unstudied subgroups or mechanisms underlying persistent symptoms	A null average treatment effect excludes persistence in every patient
Observational cohorts	Associations, symptom trajectories, risk factors, and subgroup hypotheses	Causal mechanisms or treatment efficacy	Association establishes mechanism or treatment effect
Case reports or series	Clinical heterogeneity, rare presentations, and hypothesis generation	Generalisable efficacy or prevalence estimates	Response in selected cases establishes broad treatment benefit
Animal models	Tissue-persistence, retained material, and immune-response mechanisms within defined experimental systems	Human symptom causation or clinical efficacy	Persistence in animals establishes viable infection as the cause of persistent human symptoms
*In vitro* studies	Drug tolerance, morphology, and mechanistic pathways under experimental conditions	Viable persistence in patients or safe and effective human treatment regimens	*In vitro* susceptibility or tolerance predicts clinical response
Diagnostic studies	Test performance under defined reference standards and sampling conditions	Active infection or the aetiology of persistent symptoms without appropriate clinical linkage	Test positivity establishes active infection
Guidelines	Clinical recommendations based on evidence synthesis and risk–benefit appraisal	Biological impossibility of mechanisms not directly tested	Clinical guidance resolves all mechanistic uncertainty
Reviews and meta- analyses	Synthesis of existing evidence, pooled estimates where appropriate, and identification of heterogeneity	Primary evidence or conclusions exceeding the quality and applicability of included studies	Evidence synthesis removes the limitations of the included studies
Editorials and commentaries	Interpretive framing, controversy mapping, and hypothesis articulation	Empirical treatment effects or mechanistic conclusions	Interpretive argument is treated as primary empirical evidence

The illustrative overextensions are schematic formulations used to mark inferential boundaries; they are not direct quotations from the literature.

By combining corpus-level mapping with targeted full-text appraisal, the study provides an auditable account of how evidence is translated across domains. The resulting framework supports testable questions about how mechanistic, observational, and clinical findings are connected and where additional evidence is needed to move from experimental-system-specific mechanisms to patient-level explanation or treatment evaluation.

## 5 Limitations

The corpus-level analysis used abstracts. Abstracts provide consistent coverage at scale but can omit eligibility criteria, null secondary outcomes, adverse events, caveats and mixed conclusions. The labels therefore represent abstract-level claims rather than complete reconstructions of each paper. Targeted full-text audits provided fuller evidentiary context for selected high-leverage domains but did not remove the limitations of abstract-level classification across the full corpus. Classification depended on the predefined taxonomy, prompt wording, model endpoints, available decoding settings, aggregation rules, and taskspecific handling of complete disagreement. Model provider diversity reduced reliance on one endpoint but did not eliminate correlated error. We reported pre-resolution agreement and disagreement burden across the three production models, but we did not conduct full corpus-scale sensitivity analyses using single-model outputs, unanimous-only inclusion, alternative ensembles, or alternative tie-resolution procedures. The reported distributions therefore remain conditional on the documented task-specific ensemble workflow.

The prompt-development set comprised 91 exact-agreement records inherited from the prior study rather than an independent validation sample. Conditioning on exact expert agreement favoured clearer cases, and the 19-record held-out prompt-development partition was too small for precise class-specific estimates.

The 59 expert-disagreement records lacked a single agreed target and could not support conventional accuracy, F1, or kappa without new adjudication. Independently adjudicated human labels were also unavailable for the study-design task. Model agreement does not establish correctness, and exact tier counts and odds may therefore contain unquantified classification error. Independent validation would require a prespecified study-design coding manual, stratified sampling, multiple blinded human reviewers, and formal adjudication.

Hosted-model inference has a temporal reproducibility limit. Archived inputs, prompts, original outputs, and scripts preserve the reported analysis, but a fresh run may differ after provider changes even under nominally identical settings. The workflow used temperatures of 0 or 1 depending on the task, and no seed parameter was supplied to the model calls; support for such a parameter varies across hosted endpoints and providers. The recorded settings and archived outputs improve auditability but cannot guarantee exact reproduction after provider-side changes. Stance-orientation odds are descriptive summaries of model-derived abstract labels. They are not measures of study quality, truth, treatment effect, or causal evidentiary strength. The RCT and guideline groups were small and had wide intervals, so their apparent direction should be used only to guide closer appraisal. Confidence intervals capture count-based imprecision but do not incorporate all classification uncertainty. The full-text components were bounded audits. The six-paper treatment audit comprised four retreatment or longer-term therapy trials in persistent-symptom populations and two adjacent treatment-duration trials in disseminated or late Lyme disease; it was not an exhaustive systematic review. The 24-paper preclinical set was citation-conditioned and design-balanced; it was suited to tracing evidence flow but may underrepresent less-cited null, negative, or cautious work and cannot estimate the prevalence of persistence findings. Finally, the PTLDS–CLD and study-design taxonomies compress heterogeneous positions and adjacent document categories. A paper may support persistence plausibility while rejecting prolonged treatment, or use PTLDS terminology while retaining mechanistic uncertainty. The classifications should be read as a corpus map of dominant abstract-level orientation, not a complete ontology of scientific positions.

## 6 Conclusion

This study combined prompt-optimised ensemble classification with targeted full-text appraisal to examine how claim orientations were distributed across evidence domains and how selected findings were translated into broader clinical and mechanistic arguments. Among the higher-volume tiers, observational studies and commentaries showed PTLDS-oriented distributions, whereas case reports or series, animal models, and *in vitro* studies showed CLD-oriented distributions. The smaller RCT and guideline groups were also PTLDS-oriented, but their small sizes and wide confidence intervals made these estimates too uncertain for interpretation beyond guiding closer appraisal.

The treatment audit preserved the distinct findings of individual trials while showing that their clinical interpretation depends on the durability and breadth of treatment effects, the populations enrolled, treatment burden, and safety. The preclinical audit identified experimental-system-specific findings that support several candidate mechanisms and generate hypotheses for further study, although their inferential reach varied with the experimental system and outcome measured. Together, these analyses clarify the distinction between evidence for a possible biological mechanism and evidence that the mechanism causes persistent human symptoms or identifies an effective treatment.

Future research should connect these evidence tiers more directly through measures that distinguish viable infection from retained microbial material or post-infectious immune activity, prospectively defined patient subgroups, and trials that link mechanistic eligibility criteria to clinically meaningful and durable outcomes. Such studies would test whether mechanistic signals identify clinically relevant subgroups and predict treatment response. The present study provides a structured descriptive account of how these evidentiary connections are currently represented across the PTLDS–CLD literature and where further evidence is required.

## Data Availability

Selected raw data not subject to third-party copyright, together with analysis code is available at https://github.com/teosusnjak/Lyme-disease-controversy.
