# Supplementary material for: Evidence-domain translation in the Lyme disease controversy over persistent post-treatment symptoms
Source: Front Cell Infect Microbiol. 2026 Sep 7;16:1913455. doi: 10.3389/fcimb.2026.1913455 (PMC13590328; doi:10.3389/fcimb.2026.1913455)
Supplement: Supplementary file 1 [file SupplementaryFile1.pdf]

Supplementary Material

1 SUPPLEMENTARY METHODS: AI-ASSISTED CLASSIFICATION IMPLEMENTATION

The main Methods summarise the AI-assisted classification workflow at a level intended for biomedical readers. This supplementary note records the implementation details needed for reproducibility. The human-labelled material used for stance/routing prompt development originated in the prior study; it was not generated as a new validation sample in the present study. In that earlier work, two domain experts independently classified a random sample of 150 abstracts, assigning the same label to 91 and different labels to 59. The present workflow reused the 91 fixed exact-agreement labels, divided into 54 training, 18 internal-validation, and 19 held-out prompt-development records. The internal-validation partition was used to select candidate instructions; the held-out partition was not used to choose prompt wording. The five operational labels were Supports PTLDS, Supports CLD, Neutral, Unrelated, and Animal Study. During GEPA optimisation, the feedback metric was binary: a prediction received a score of 1 when it matched the inherited reference label and 0 otherwise. The 59 disputed records lacked a single agreed human target and were not scored as a conventional test set. Because the 91 records were few and conditioned on exact expert agreement, their results support prompt development but do not provide a precise estimate of corpus-wide post-optimisation validity.

Candidate stance/routing experiments compared multiple LLMs and prompt starting points. The model set included `gpt-oss-20b`, `gpt-4o-mini`, `gpt-5-mini`, Gemini 2.0 Flash, and Grok-4 Fast. Initial instruction variants included a minimal classification directive, the prior-study prompt, and a shorter prompt consisting primarily of class descriptions plus a classification instruction. GEPA refined natural-language instructions without changing model weights or label definitions and was run with a medium automatic optimisation budget. Final corpus labelling did not rely on a single model output: `gpt-5-mini`, Grok-4 Fast, and Gemini 2.0 Flash were selected using development-set performance together with provider diversity, availability, structured-output suitability, and operational feasibility. Each model classified a record independently under the same operational definitions and without access to the other outputs. A two-of-three majority determined the model-derived stance/routing label when at least two models agreed. Complete three-way stance/routing disagreements entered a separate prompt-based adjudication stage using the source abstract, the three candidate labels, and their short rationales. The adjudication call used `openrouter/x-ai/grok-4-fast`, thereby reusing one of the production model endpoints in a distinct role rather than adding a fourth model. It applied the same predefined definitions and returned a label with a justification. This was a model-based procedure, not ad hoc human adjudication.

The adjudicator endpoint for stance/routing ensemble was set at temperature of 1, and no supplied seed parameter was used. For primary study design, the local aggregation code assigned the sentinel value `nothing_matched` when all three primary labels differed; these records did not enter a further model call. The thematic task used a separate multilabel consensus rule. The workflow therefore did not apply one universal adjudication procedure across all classification tasks.

For theme labelling, the inherited eight-theme taxonomy was first checked for adequacy in the reclassified debate-relevant subset. Debate-relevant abstracts were batched and submitted to the ensemble models with instructions to identify whether recurrent themes were missing from the inherited taxonomy. Candidate

additions were consolidated and compared across models. This check did not identify a robust cross-model need to replace the inherited taxonomy, so the original eight themes were retained. For final thematic assignment, each model generated candidate theme labels for each abstract. Retained themes were determined using the strict cross-model consensus rule described in the main Methods, which was selected to avoid inflated theme prevalence under looser voting rules.

For study-design and evidence-domain labelling, the taxonomy was predefined before corpus application and allowed one primary study-design label plus secondary labels where applicable. It described the design reported in the title and abstract; it did not assess study quality or risk of bias. Because independently adjudicated human study-design labels were not available at corpus scale, prompt development used a pragmatic high-confidence model-consensus subset: records for which `gpt-5-mini`, Grok-4 Fast, and Gemini 2.0 Flash agreed on the primary label. These consensus labels were used only to stabilise prompt development. They were not treated as an independent human reference standard. In the production aggregation, a two-of-three majority determined the primary label; complete three-way disagreements were retained as `nothing_matched`. Corpus-wide study-design and evidence-domain labels therefore remain model-derived and were not independently human validated in this revision.

The analysis has retained the available endpoint identifiers, compiled prompts, operational definitions, generation settings recorded by the workflow, task-specific aggregation and disagreement-resolution rules, original outputs, derived labels and analysis scripts to be made available online. The experimental runs were completed in November 2025; record temperatures of 0 or 1 depending on the task, no seed parameter was supplied to model calls, and JSON was the structured-output format. Seed-parameter support varies across hosted endpoints and providers; the absence of a supplied parameter is reported as an implementation fact. These materials support reconstruction of the experimental analysis but cannot guarantee identical future outputs from hosted endpoints, whose weights, aliases, routing, system instructions, safety layers, or serving infrastructure may change.

Alternative candidate models were compared during prompt development. A complete corpus-scale comparison of each production model, unanimous-only aggregation, alternative tie-resolution rules, and every available endpoint was not performed. The reported estimates are conditional on the documented task-specific ensemble procedures. Independent human validation of study-design labels and alternative-aggregation sensitivity analyses remain priorities for a separately planned validation study.

Table S1: Pre-resolution agreement and complete-disagreement burden across the three production models

**Agreement measure**	**Stance/routing**	**Primary study design**
Number of records	8,630	1,373
Unanimous agreement, n (%)	5,926 (68.7)	1,011 (73.6)
Two-to-one agreement, n (%)	2,560 (29.7)	325 (23.7)
Three-way disagreement, n (%)	144 (1.7)	37 (2.7)
Complete three-way disagreement, n (%)	144 (1.7)	37 (2.7)
Pairwise agreement (%)	84.6 / 77.5 / 73.6	83.5 / 80.6 / 80.6
Fleiss' κ	0.629	0.789

Note: Agreement was calculated from the three experimental pre-resolution output files, aligned by record order and verified against identical abstract text at each position. Pairwise percentages are reported in the order `gpt-5-mini`–Grok-4 Fast, `gpt-5-mini`–Gemini 2.0 Flash, and Grok-4 Fast–Gemini 2.0 Flash. Fleiss' κ measures inter-model consistency, not validity against a human reference standard. The 1,373 study-design records were the debate-relevant inputs available at that workflow stage. Record-level reconciliation found that three workflow-stage entries were absent from the final debate-relevant file and one duplicate entry appeared only in the final integrated file, producing the final 1,371-record evidence-domain dataset. For study design, one missing primary label from the `gpt-5-mini` output was retained as "Not classified." Theme assignment used a multilabel consensus rule and is not summarised by this mutually exclusive-label table. The 144 stance/routing disagreements entered model-based adjudication using `openrouter/x-ai/grok-4-fast`; the 37 primary study-design disagreements were retained by the local aggregation code as `nothing_matched` and were not model-adjudicated.

Table S2: Verified run metadata and documented gaps in the study workflow. Temperature was set to 0 or 1 depending on the task; no seed parameter was supplied to model calls; and structured outputs were returned in JSON format. Seed-parameter support varies across hosted endpoints and providers.

**Task**	**Model/provider**	**Recorded endpoint string or model identifier**	**Run date or range**	**Role**
Stance/routing; theme; study design	OpenAI	`gpt-5-mini`	Nov. 2025	Production classifier
Stance/routing; theme; study design	xAI via OpenRouter	`x-ai/grok-4-fast`	Nov. 2025	Production classifier
Stance/routing; theme; study design	Google via OpenRouter	`google/gemini-2.0-flash-001`	Nov. 2025	Production classifier
Stance/routing complete disagreement	xAI via OpenRouter	`openrouter/x-ai/grok-4-fast`	Nov. 2025	Model-based adjudication; temperature 1
Primary study-design complete disagreement	Local aggregation code	`nothing_matched` (no endpoint call)	Nov. 2025	Unresolved sentinel assignment
GEPA development	OpenAI	`gpt-oss-20b`	Nov. 2025	Candidate student and reflector
GEPA development	OpenAI	`gpt-4o-mini`	Nov. 2025	Candidate student or reflector
GEPA development	OpenAI	`gpt-5-mini`	Nov. 2025	Candidate student or reflector
GEPA development	xAI via OpenRouter	`x-ai/grok-4-fast`	Nov. 2025	Candidate student and reflector
GEPA development	Google via OpenRouter	`google/gemini-2.0-flash-001`	Nov. 2025	Candidate student and reflector

2 SUPPLEMENTARY EVIDENCE-EXTRACTION AND EVIDENCE-FLOW TABLES

Supplementary Tables S1 and S2 report the classification-workflow audit: pre-resolution agreement, complete-disagreement burden, recoverable endpoint strings, task-specific resolution procedures, and documented metadata gaps. Supplementary Tables S3–S9 provide the audit trail for the targeted full-text components. They record the structured clinical-trial extraction and the preclinical evidence-flow audit,

including the citation-overlap denominator flow, eligible source-conditioning documents, full-manuscript audit of flagged source labels, selected 24-paper preclinical extraction subset, mechanism and evidence-translation boundary coding, and influential cited candidates that were not included in the final extraction subset.

2.1 Clinical trial extraction

Supplementary Table S3 provides the structured full-text extraction basis for the retreatment and adjacent treatment-duration trials discussed in the main text. From 13 retained clinical candidates, the detailed appraisal selected four trials directly evaluating retreatment or longer-term therapy in persistent-symptom populations and two adjacent randomised duration trials in disseminated or late Lyme disease. Protocols, commentaries, secondary or biomarker reports, coinfection-specific interventions, and acute erythema-migrans duration studies were not included in the six-trial appraisal. The table records population eligibility, intervention and comparator, primary or main result, follow-up and safety information, and the defensible evidentiary translation for each trial. Its purpose is to support the clinical-utility interpretation of the RCT evidence rather than to function as an additional meta-analysis.

Table S3: Structured appraisal fields for retreatment and adjacent treatment-duration trials

Study / design / n	Population and eligibility	Intervention and comparator	Primary or main result	Follow-up and safety	Applicability and defensible translation
Klempner et al. (2001); two parallel randomized placebo-controlled trials; 129 enrolled	Previously treated, well-documented Lyme disease with persistent symptoms; separate seropositive and seronegative cohorts	IV ceftriaxone for 30 days followed by oral doxycycline for 60 days versus matched placebo	No significant antibiotic advantage on SF-36 physical or mental component outcomes at day 180; trials stopped after interim futility analysis	Main endpoint at 180 days; study-related adverse events occurred in both groups, with serious treatment-related events in antibiotic recipients	Directly relevant to documented post-treatment persistent symptoms; does not support routine prolonged retreatment, while not testing every possible mechanism-defined subgroup
Krupp et al. (2003); randomized double-masked placebo-controlled trial; 55 randomized	Post-Lyme syndrome with severe fatigue at least six months after prior antibiotics; documented erythema migrans or CDC late Lyme disease with positive ELISA and Western blot	IV ceftriaxone for 28 days versus IV placebo	Fatigue improved significantly with ceftriaxone; no corresponding cognitive benefit or CSF OspA clearance signal	Six-month assessment; treatment-associated hospitalizations occurred in both arms	Supports a fatigue-specific signal in a selected documented population; insufficient alone for broad retreatment efficacy or persistent-infection mechanism claims
Fallon et al. (2008); randomized placebo-controlled trial; 37 patients	Previously treated Lyme encephalopathy; well-documented prior Lyme disease, current positive IgG Western blot, and objective memory impairment	IV ceftriaxone for 10 weeks versus IV placebo	Cognitive improvement at week 12, with relapse after antibiotics stopped; some secondary pain and physical-function signals persisted	Follow-up to week 24; medication or PICC-line adverse events were more frequent with ceftriaxone, without permanent injury reported	Relevant to a narrow neurocognitive subgroup; supports short-term treatment responsiveness but not durable cognitive strategy or proof of viable infection
Berende et al. (2016); randomized double-blind placebo-controlled trial; 280 modified intention-to-treat participants	Persistent symptoms attributed to Lyme disease, temporally related to proven Lyme disease or accompanied by positive IgG or IgM immunoblot evidence	All groups received two weeks open-label IV ceftriaxone, then 12 weeks doxycycline, clarithromycin–hydroxychloroquine, or placebo	No significant difference among groups on RAND SF-36 physical-component score at end of treatment; all groups improved from baseline	Follow-up to 52 weeks; serious drug-related events occurred during open-label ceftriaxone, with no serious drug-related event during randomized oral therapy	Weighs against longer-term oral antibiotic therapy after initial ceftriaxone under the study criteria; does not adjudicate untested biomarker-defined subgroups

Table S3: Structured appraisal fields for retreatment and adjacent treatment-duration trials (continued)

Study / design / n	Population and eligibility	Intervention and comparator	Primary or main result	Follow-up and safety	Applicability and defensible translation
Oksi et al. (2007); double-blind randomized placebo-controlled treatment-extension trial; final analysis 145 patients	Disseminated Lyme borreliosis classified as definite or possible using clinical manifestations and laboratory evidence	Both arms received IV ceftriaxone for 21 days, followed by oral amoxicillin or placebo for 100 days	Outcomes after one-year follow-up did not show a statistically significant advantage for adjunct oral amoxicillin; authors did not justify oral extension after ceftriaxone	Follow-up at 1, 3, 6, and 12 months; no serious antibiotic adverse effects, but diarrhea was common and rare C. difficile events occurred	Adjacent duration evidence for disseminated Lyme borreliosis; informative for extension claims but not a direct PTLDS–CLD retreatment trial
Dattwyler et al. (2005); open-label randomized multicentre trial; 201 randomized, 143 seropositive efficacy recipients	Late Lyme disease with objective neurologic, rheumatologic, or dermatologic manifestations; efficacy analysis restricted to patients meeting ELISA and Western blot criteria	Ceftriaxone for 14 days versus 28 days	Clinical cure rates at last evaluation were similar; more failures occurred in the 14-day group, but the trial was not powered to define responder subgroups	Follow-up through post-treatment visits; discontinuation due to adverse events was significantly more frequent with 28 days	Adjacent late-Lyme duration evidence; supports caution about extending ceftriaxone routinely while illustrating how serological eligibility narrows trial applicability

2.2 Preclinical citation-overlap sampling frame

The preclinical audit used eligible guideline, review, and meta-analysis documents as source-conditioning sources to identify influential preclinical studies cited in evidence-synthesis and guidance literature. One case report initially assigned a guideline label by the model-derived workflow was removed after publication-type verification. Supplementary Table S4 summarises the denominator flow from source documents and extracted reference strings to matched preclinical targets, the selected 24-paper extraction subset, and the unmatched-candidate sensitivity checks. Supplementary Table S5 lists the 53 eligible source-conditioning documents used for this citation-overlap sampling step.

Table S4. Preclinical citation-overlap and unmatched-candidate sensitivity audit summary

**Audit component**	***n***	**Interpretive use**
Eligible guideline, review, and meta-analysis source documents accounted for	53	Publication-type-verified source-conditioning frame after removal of one misclassified case report
Extracted reference strings from available full-text conversions	4,304	Reference pool used for DOI and title/year matching
Preclinical reference matches	147	Matched evidence-flow links from guideline/review sources to corpus-derived preclinical targets
Unique cited preclinical targets	70	Candidate pool before selecting the 24-paper extraction subset
Selected extraction subset	24	Design-balanced full-text synthesis set: 12 animal/in vivo and 12 *in vitro* studies
High-value unmatched candidate rows triaged	441	Sensitivity audit trail for records with persistence, persister, biofilm, xenodiagnosis, animal, *in vitro*, culture, cystic, round-body, or antibiotic-treatment terms
Manual-override candidates preserved but not promoted	100	Candidate records retained for transparency but not used to override the prespecified target frame

The audit supports the use of the 24-paper subset as an influential, citation-conditioned evidence-flow sample. It does not make the subset statistically representative of all preclinical Lyme disease research.

Table S5: Eligible guideline, review, and meta-analysis source documents used for citation-overlap sampling

No.	Citation	Year	Stance
1	Jaulhac et al. (2019)	2019	Supports PTLDS
2	Gaubitz et al. (2014)	2014	Supports PTLDS
3	Shor et al. (2019)	2019	Supports CLD
4	Rauer et al. (2018)	2018	Supports PTLDS
5	Zimmerli (2016)	2016	Supports PTLDS
6	Shapiro (2014)	2014	Supports PTLDS
7	Cameron et al. (2014)	2014	Supports CLD
8	Mygland et al. (2010)	2010	Supports PTLDS
9	Bever et al. (2007)	2007	Supports PTLDS
10	Wormser et al. (2006)	2006	Supports PTLDS
11	Miklossy (2011)	2011	Supports CLD
12	Kraiczy (2016)	2016	Supports CLD
13	Motamed et al. (2023)	2023	Supports CLD
14	Custodio et al. (2022)	2022	Neutral
15	Dumler (2001)	2001	Neutral
16	Dersch and Rauer (2023)	2023	Supports PTLDS
17	Aberer and Wutte (2016)	2016	Supports CLD
18	Zhang et al. (2023)	2023	Supports CLD
19	Gueye et al. (2019)	2019	Supports CLD
20	Stinco et al. (2016)	2016	Neutral
21	Zadeh (2007)	2007	Supports PTLDS
22	Jakubowska et al. (2024)	2024	Neutral
23	Dersch et al. (2024)	2024	Supports PTLDS
24	Bashchobanov et al. (2024)	2024	Neutral
25	Bockenstedt and Belperron (2024)	2024	Neutral
26	Strzoda et al. (2023)	2023	Supports PTLDS
27	Adkison and Embers (2023)	2023	Neutral
28	Guérin et al. (2023)	2023	Supports CLD
29	Bai and Richardson (2023)	2023	Supports PTLDS
30	Talbot et al. (2023)	2023	Supports PTLDS
31	Skripchenko et al. (2022)	2022	Supports CLD
32	Wong et al. (2022)	2022	Neutral
33	Hernandez-Ruiz et al. (2022)	2022	Neutral
34	Verschoor et al. (2022)	2022	Supports PTLDS
35	Thompson et al. (2021)	2021	Neutral
36	Woitzik and Linder (2021)	2021	Neutral
37	Raffetin et al. (2020)	2020	Neutral
38	Rebman and Aucott (2020)	2020	Neutral
39	Brouwer et al. (2020)	2020	Supports PTLDS
40	Jarosz and Badawi (2019)	2019	Supports PTLDS
41	Aslam et al. (2017)	2017	Supports CLD
42	Verhaegh et al. (2017)	2017	Supports CLD
43	Hyde (2017)	2017	Supports CLD
44	Tracy and Baumgarth (2017)	2017	Supports CLD
45	Zubair Alam et al. (2017)	2017	Supports CLD
46	Lin et al. (2017)	2017	Supports CLD
47	Goc and Rath (2016)	2016	Supports CLD
48	Chalada et al. (2016)	2016	Neutral

Continued on next page

Table S5: Eligible guideline, review, and meta-analysis source documents used for citation-overlap sampling (continued)

No.	Citation	Year	Stance
49	Bankhead (2016)	2016	Supports CLD
50	Bogdos et al. (2016)	2016	Neutral
51	Borgermans et al. (2014)	2014	Neutral
52	Miklossy (2012)	2012	Supports CLD
53	Embers et al. (2004)	2004	Supports CLD

Note: Stance labels in this source-conditioning table are model-derived labels used to structure citation-overlap sampling, not independent determinations of evidentiary validity or full guideline position.

2.3 Full-manuscript audit of flagged source-conditioning labels

Some source-conditioning records had ambiguous or potentially unstable abstract-level orientations. Supplementary Table S6 reports a targeted full-manuscript audit of these flagged records. The table distinguishes the abstract-level model-derived label from the full-manuscript audited label, so differences between the two should be read as source-conditioning refinements rather than as simple model errors. Its purpose is to document how borderline, mixed, diagnostic, or adjacent sources were handled before they were used in the preclinical evidence-flow sampling frame.

Table S6: Targeted full-manuscript audit of flagged abstract-level source-conditioning labels

Citation	Abstract-level model-derived label	Full-manuscript audited label	Audit rationale
Zhang et al. (2023)	Supports CLD	Supports CLD	The abstract supports the model-derived label because it reports a network meta-analysis in which ceftriaxone performs best on fatigue-severity outcomes and concludes that ceftriaxone may be the best antibiotic option for PTLD. Full-manuscript review confirms a CLD-oriented source-conditioning role, but only in a narrow treatment-signal sense: the positive finding is centred on fatigue and comparative antibiotic ranking, while other outcomes show limited or no significant advantage. The paper does not provide a direct viable-persistence claim, so it should be treated as treatment-positive evidence within the controversy rather than as mechanistic proof of persistent infection.
Dersch et al. (2024)	Supports PTLDS	Supports PTLDS	The abstract already gives a clear PTLDS-aligned orientation: the review reports no statistically significant antibiotic-placebo difference for quality of life, cognition, or depression; inconsistent fatigue results; and more adverse events with antibiotics. Full-manuscript review confirms this source-conditioning role. The paper is organised around evidence-based treatment guidance for post-treatment symptoms and concludes that antibiotics do not show sufficient net benefit for suspected PTLDS.

Table S6: Targeted full-manuscript audit of flagged abstract-level source-conditioning labels (continued)

Citation	Abstract-level model-derived label	Full-manuscript audited label	Audit rationale
Adkison and Embers (2023)	Neutral	Supports CLD	The abstract makes the model-derived Neutral label understandable because it presents several competing explanations for treatment failure, including autoimmune responses, long-term sequelae from initial infection, and spirochetal persistence, and states that the review considers evidence that both validates and challenges these mechanisms. Full-manuscript review, however, shows a more persistence-friendly source-conditioning role: the paper foregrounds treatment failure, immune evasion, possible surviving spirochetes, biomarkers, and the need for definitions and guidelines to evolve with mechanistic research. The audited label therefore treats the source as CLD-supporting for evidence-flow purposes, while recognising that it is a mechanism-oriented and mixed-causal review rather than a direct claim that prolonged antibiotic therapy is clinically effective.
Guérin et al. (2023)	Supports CLD	Supports CLD	The abstract supports the model-derived label because it emphasises limitations of standard two-tier serology, low sensitivity in some disease stages, underdiagnosis or misdiagnosis, therapeutic wandering, possible chronic infection, and the need for early detection to avoid pathogen persistence. Full-manuscript review confirms this orientation. The source is best interpreted as CLD-supporting in a diagnostic and persistence-oriented sense, because it argues for improved detection and active-infection discrimination, not as evidence that prolonged antibiotic treatment is clinically effective.
Talbot et al. (2023)	Supports PTLDS	Neutral	The abstract makes the model-derived PTLDS label understandable because it uses PTLDS terminology, presents PTLDS as a post-treatment syndrome of uncertain aetiology, and frames available PTLDS care as sparse and largely underdeveloped rather than as established evidence for chronic infection. Full-manuscript review shows that the source is more mixed than a single PTLDS-supporting label implies. It discusses limited evidence for latent infection and notes that antibiotic studies have not shown significant symptom relief, but it also reviews lingering or drug-resistant *B. burgdorferi*, autoimmune, peptidoglycan, central-sensitisation, vaccine, and emerging antibiotic hypotheses. The audited label is therefore Neutral because the paper functions as a broad treatment-options review rather than a clean PTLDS- or CLD-aligned source.
Wong et al. (2022)	Neutral	Supports PTLDS	The abstract explains why a model-derived Neutral label was plausible: it presents the article as a review of both PTLDS and chronic Lyme disease, including diagnostic testing, persistence hypotheses, immune dysregulation, protracted symptoms, and IVIG. Full-manuscript review clarifies a stronger PTLDS-aligned stance. The paper explicitly distinguishes PTLDS from the broader and poorly defined category of chronic Lyme disease, states that chronic Lyme disease is not convincingly attributable to persistent *B. burgdorferi* infection, and concludes that additional antibiotics or IVIG are not supported for persistent symptoms attributed to Lyme disease. The audited label therefore treats the source as PTLDS-supporting.
Verschoor et al. (2022)	Supports PTLDS	Supports PTLDS	The abstract and full manuscript align. The review appraises experimental animal evidence for persistent *Borrelia burgdorferi sensu lato* infection after antibiotic treatment and acknowledges that some animal studies report sporadic post-treatment culture or nucleic-acid findings. However, the full manuscript concludes that available animal studies provide insufficient evidence for viable and infectious spirochetes after adequate antibiotic treatment, and that post-treatment DNA or antigen detection should not be equated with clinically relevant viable infection. The source therefore supports a PTLDS-oriented evidentiary boundary around animal persistence claims.

Table S6: Targeted full-manuscript audit of flagged abstract-level source-conditioning labels (continued)

Citation	Abstract-level model-derived label	Full-manuscript audited label	Audit rationale
Miklossy (2011)	Supports CLD	Supports CLD-adjacent	The model-derived CLD label is supportable from the abstract because the paper argues for chronic spirochetal involvement in Alzheimer disease, reports *Borrelia burgdorferi* detection in a subset of Alzheimer disease cases, and frames neurospirochetosis, chronic inflammation, and amyloid deposition as potentially linked through chronic infection. Full-manuscript review confirms the persistence-oriented reasoning but narrows the source-conditioning role. The paper is primarily an Alzheimer disease neurospirochetosis review, not a PTLDS–CLD treatment, guideline, or persistent post-treatment symptom paper. It is therefore retained as CLD-adjacent rather than as a direct CLD source.
Miklossy (2012)	Supports CLD	Supports CLD	The abstract and full manuscript align. The paper directly addresses chronic or late Lyme neuroborreliosis, compares it with chronic neurosyphilis, and argues that *Borrelia burgdorferi* can evade host immune destruction, persist in host tissues, and sustain chronic infection and inflammation. Because the source directly links late/chronic Lyme neuroborreliosis to persistent spirochetal infection, the full-manuscript audit confirms the CLD-supporting label.
Dumler (2001)	Neutral	Neutral	The abstract supports the model-derived Neutral label because the review is focused on molecular diagnosis rather than on adjudicating PTLDS versus CLD. It emphasises assay variability, limited clinical validation, low sensitivity in some specimen types, and the persistence of *B. burgdorferi* DNA in urine or synovial fluid after therapy and convalescence. Full-manuscript review confirms that the source supports diagnostic caution: persistent DNA is treated as a limitation for primary diagnosis and disease monitoring, not as proof of viable infection or as evidence against persistence-oriented claims. The audited label therefore remains Neutral.

Note: The abstract-level model-derived label reflects classification based on the article abstract and bibliographic metadata. The full-manuscript audited label reflects manual review of the full source and its role in the preclinical citation-overlap source-conditioning audit. Differences between the two labels do not necessarily indicate model error; in some cases, the abstract supports a defensible broad orientation while the full manuscript clarifies a narrower, mixed, or adjacent role. "CLD-adjacent" denotes a persistence-oriented source relevant to CLD-style reasoning but not primarily focused on PTLDS–CLD treatment, guideline reasoning, or persistent post-treatment symptoms.

2.4 Selected preclinical extraction subset

Supplementary Table S7 reports the selected 24-paper preclinical extraction subset and the citation-overlap justification for each included paper. The table shows how papers were selected from the cited preclinical target pool using citation uptake, guideline/review use, design balance, and mechanism coverage. It should be read as a transparent rationale for a targeted evidence-flow sample, not as a claim that the selected papers are statistically representative of all preclinical Lyme disease research.

Table S7: Selected 24-paper preclinical extraction subset and citation-overlap justification

No.	Citation	Rank	Citing records	Guideline cites	DOI-exact matches	All matches	Selection basis
1	Hodzic et al. (2008)	1	7	2	2	7	Top-cited murine antibiotic-treatment persistence model; central active-infection/therapy controversy anchor.

Continued on next page

Table S7: Selected 24-paper preclinical extraction subset and citation-overlap justification (continued)

No.	Citation	Rank	Citing records	Guideline cites	DOI-exact matches	All matches	Selection basis
2	Embers et al. (2012)	2	7	2	0	7	Top-cited non-human-primate antibiotic-treatment persistence model; high translational salience.
3	Bockenstedt et al. (2012)	3	6	1	2	6	Highly cited murine post-therapy antigen-persistence paper; bridges persistence and immune-positioning claims.
4	Liang et al. (2004)	5	5	0	2	5	Highly cited protective-niche animal evidence for immune evasion and tissue persistence.
5	Liang et al. (2002)	8	4	2	1	4	Influential immune-evasion/spirochetal-persistence animal paper cited by guideline/review records.
6	Jutras et al. (2019)	10	4	1	1	4	Persistent peptidoglycan antigen paper; important PTLDS/post-infectious immune mechanism comparator.
7	Crossland et al. (2018)	20	2	0	2	2	Later post-treatment pathology and spirochete-persistence model; adds contemporary animal-pathology evidence.
8	Elsner et al. (2015)	27	2	0	1	2	Suppression of long-lived humoral immunity after infection; captures immune dysregulation mechanism.
9	Yrjänäinen et al. (2010)	30	2	0	1	2	Borrelial DNA persistence after ceftriaxone in mice; treatment-response persistence stratum.
10	Behera et al. (2005)	32	2	0	1	2	Neutral/immune-positioning animal paper on host matrix metalloproteinases; protects against a purely CLD-supportive sample.
11	Yrjänaäinen et al. (2007)	36	2	0	0	2	Anti-TNF reactivation after ceftriaxone model; explicit therapy and immune-suppression relevance.
12	Chang et al. (2001)	68	1	0	0	1	Dog chronic-borreliosis model; lower-ranked but retained for in vivo host-species/design diversity.
1	Miklossy et al. (2008)	4	6	0	1	6	Top-cited atypical/cystic-form and neuroinflammation paper; morphology/persistence anchor.
2	Livengood and Gilmore Jr (2006)	6	4	2	1	4	Neuronal/glial cell invasion paper; neuroborreliosis and intracellular invasion mechanism.
3	Kraiczy et al. (2004)	7	4	2	1	4	Complement resistance/BbCRASP-1 paper; immune-evasion anchor.
4	Sharma et al. (2015)	9	4	1	2	4	Drug-tolerant persister-cell paper; central in vitro persistence/therapy evidence.
5	Hartiala et al. (2007)	11	3	2	1	3	Human dendritic-cell transcriptional response; immune dysregulation stratum.
6	Feng et al. (2015)	12	3	1	2	3	In vitro drug-combination eradication paper; treatment-efficacy controversy stratum.
7	Feng et al. (2014)	13	3	1	2	3	FDA-library screen for persisters; high-throughput treatment-discovery stratum.
8	Pothineni et al. (2020)	14	3	0	1	4	Azlocillin/drug-tolerant Borrelia paper; later treatment-discovery evidence.
9	Iyer et al. (2013)	15	3	0	1	3	Nucleic-acid detection after antibiotics does not confirm viability; PTLDS/diagnostic caution comparator.
10	Sapi et al. (2011)	16	3	0	1	3	Morphological-form antibiotic susceptibility paper; cyst/round-body therapy stratum.
11	Alitalo et al. (2002)	17	3	0	1	3	Factor H binding/complement evasion paper; immune-evasion mechanism.

Continued on next page

Table S7: Selected 24-paper preclinical extraction subset and citation-overlap justification (continued)

No.	Citation	Rank	Citing records	Guideline cites	DOI-exact matches	All matches	Selection basis
12	Feng et al. (2016)	18	2	1	1	2	Amoxicillin-induced round-body drug screen; morphology plus drug-tolerance stratum.

2.5 Mechanism and evidence-translation boundary coding

Supplementary Table S8 provides the structured full-text coding for the selected preclinical papers. For each paper, it records the mechanism, model or system, treatment context, supported viability inference, and evidence-translation boundary. This table is the main provenance record for the manuscript's claim that preclinical persistence studies provide model-bound support for candidate mechanisms more strongly than direct clinical treatment claims.

Table S8: Mechanism and evidence-translation boundary coding for the selected preclinical papers

Citation	Mechanism	Model/system	Treatment context	Supported viability inference	Translation boundary
Hodzic et al. (2008)	animal post antibiotic persistence	C3H mice experimentally infected with Borrelia burgdorferi	yes	inferred or unresolved viable persistence	Does not prove antibiotic-responsive persistent infection in human PTLDS/CLD.
Embers et al. (2012)	nonhuman primate post antibiotic persistence	Rhesus macaques with disseminated Borrelia burgdorferi infection	yes	suggestive viability not culture equivalent	Does not establish prevalence, symptom causation, or retreatment efficacy in human PTLDS/CLD.
Bockenstedt et al. (2012)	post infectious antigen persistence	Murine Lyme borreliosis model with tissue antigen localization	yes	antigen persistence not viability	Does not support living persistent organisms or antibiotic-responsive symptoms.
Liang et al. (2004)	immune evasion or tissue niche	Murine Borrelia burgdorferi infection and tissue niche model	no	viable persistence during active infection	Does not test post-treatment persistence or retreatment benefit.
Liang et al. (2002)	immune evasion or antigenic variation	Murine spirochetal infection model	no	viable persistence during active infection	Does not directly address post-treatment human disease.
Jutras et al. (2019)	post infectious antigen persistence	Human Lyme arthritis samples with experimental characterization of Borrelia peptidoglycan	yes or refractory context	antigen persistence not viability	Does not prove living organisms or antibiotic benefit.
Crossland et al. (2018)	nonhuman primate post antibiotic pathology	Rhesus macaques with late disseminated Lyme disease	yes	suggestive viability requires caution	Does not establish human symptom causation, prevalence, or retreatment efficacy.
Elsner et al. (2015)	immune dysregulation	Mouse Borrelia burgdorferi infection model	no	immune dysregulation not viability assay	Does not identify viable persistence or treatment failure as the cause of human symptoms.
Yrjänäinen et al. (2010)	animal post antibiotic molecular persistence	Borrelia burgdorferi-infected mice	yes	dna persistence viability unresolved	DNA detection alone cannot prove viable infection or treatment indication.

Continued on next page

Table S8: Mechanism and evidence-translation boundary coding for the selected preclinical papers (continued)

Citation	Mechanism	Model/system	Treatment context	Supported viability inference	Translation boundary
Behera et al. (2005)	immune pathology or tissue damage	Human and murine Lyme arthritis tissue/cell models	partial human persistent arthritis context	immune pathology not viability assay	Does not adjudicate viable persistence or antibiotic retreatment efficacy.
Yrjänaäinen et al. (2007)	animal post antibiotic reactivation	C3H/He mice infected with Borrelia burgdorferi	yes	culture reactivation in mouse after immunomodulation	Does not justify routine human retreatment or show that ordinary PTLDS is driven by the same mechanism.
Chang et al. (2001)	animal chronic infection or immunosuppression	Adult dogs exposed to Borrelia burgdorferi-infected ticks	no	chronic active borreliosis model	Does not test post-antibiotic human PTLDS or retreatment.
Miklossy et al. (2008)	morphological or cystic forms	Human Lyme neuroborreliosis tissue and in vitro Borrelia morphological observations	unclear	morphology persistence viability unresolved	Morphology alone does not prove viable antibiotic-responsive infection.
Livengood and Gilmore Jr (2006)	cellular invasion or tropism	Human neuronal and glial cells exposed to infectious Borrelia burgdorferi	no	cell invasion not post treatment viability	Does not prove chronic treated infection in humans.
Kraiczy et al. (2004)	complement resistance	Borrelia burgdorferi complement-resistance protein assays	no	immune evasion mechanism not post treatment viability	Does not establish PTLDS/CLD causation or retreatment efficacy.
Sharma et al. (2015)	drug tolerant persister cells	In vitro Borrelia burgdorferi stationary-phase/persister cultures	yes in vitro antibiotic exposure	in vitro drug tolerant persisters	Does not prove human persistent infection or clinical benefit of retreatment.
Hartiala et al. (2007)	immune dysregulation	Human dendritic cells stimulated/infected with Borrelia garinii	no	immune response not viability assay	Does not address viable persistence or treatment failure.
Feng et al. (2015)	drug tolerant persister cells	In vitro Borrelia burgdorferi persister cultures	yes in vitro antibiotic exposure	in vitro drug tolerant persisters	Does not establish safe or effective human retreatment.
Feng et al. (2014)	drug tolerant persister cells	In vitro Borrelia burgdorferi persister/drug-screening system	yes in vitro drug screen	in vitro drug tolerant persisters	Screening hits require pharmacologic and clinical validation.
Pothineni et al. (2020)	drug tolerant persister cells	In vitro drug-tolerant Borrelia burgdorferi sensu stricto with supporting model evidence	yes in vitro drug exposure	in vitro drug tolerant persisters	Does not prove clinical efficacy or safety in PTLDS/CLD.
Iyer et al. (2013)	diagnostic caution viability	Borrelia burgdorferi experimental antibiotic-treatment detection model	yes	nucleic acid detection does not confirm viability	Does not prove persistence never occurs.
Sapi et al. (2011)	morphological forms or round bodies	In vitro Borrelia burgdorferi morphological forms	yes in vitro antibiotic exposure	morphology associated drug tolerance in vitro	Does not define human treatment protocols.
Alitalo et al. (2002)	complement resistance	Borrelia spirochete complement factor H binding assays	no	immune evasion mechanism not post treatment viability	Mechanism alone is not evidence of post-treatment viable infection.

Continued on next page

Table S8: Mechanism and evidence-translation boundary coding for the selected preclinical papers (continued)

Citation	Mechanism	Model/system	Treatment context	Supported viability inference	Translation boundary
Feng et al. (2016)	morphological forms or round bodies	In vitro amoxicillin-induced Borrelia burgdorferi round bodies	yes in vitro antibiotic exposure	in vitro round body drug tolerance	Does not demonstrate human clinical efficacy.

2.6 Influential excluded preclinical candidates

Supplementary Table S9 lists influential cited preclinical targets that were not included in the 24-paper extraction subset. This table provides a selection-boundary audit: it shows that additional cited candidates were considered and preserved for transparency, while clarifying that the final extraction subset was bounded by the prespecified design-balanced and citation-conditioned sampling strategy.

Table S9: Influential cited preclinical targets excluded from the 24-paper extraction subset

Rank	Paper	Year	Design	Citing records	Guideline cites
19	Immune evasion of Borrelia burgdorferi: insufficient killing of the pathogens by complement and antibody.	2002	in_vitro	2	1
21	Identifying Vancomycin as an Effective Antibiotic for Killing Borrelia burgdorferi.	2018	in_vitro	2	0
22	Selective Essential Oils from Spice or Culinary Herbs Have High Activity against Stationary Phase and Biofilm Borrelia burgdorferi.	2017	in_vitro	2	0
23	Identification of new drug candidates against Borrelia burgdorferi using high-throughput screening.	2016	in_vitro	2	0
24	Binding of the complement inhibitor C4b-binding protein to Lyme disease Borreliae.	2010	in_vitro	2	0
25	Differential expression of matrix metalloproteinases and cyclooxygenases in synovial cells exposed to Borrelia burgdorferi	2004	in_vitro	2	0
26	Effectiveness of Stevia Rebaudiana Whole Leaf Extract Against the Various Morphological Forms of Borrelia Burgdorferi in Vitro.	2015	in_vitro	2	0
28	Microarray analyses of inflammation response of human dermal fibroblasts to different strains of Borrelia burgdorferi sensu stricto.	2012	in_vitro	2	0
29	Invasion of eukaryotic cells by Borrelia burgdorferi requires $\beta(1)$ integrins and Src kinase activity.	2011	in_vitro	2	0
31	Borrelia burgdorferi RevA antigen binds host fibronectin.	2009	in_vitro	2	0
33	Persister Development by Borrelia burgdorferi Populations In Vitro.	2015	in_vitro	2	0
34	Characterization of unique regions of Borrelia burgdorferi surface-located membrane protein 1.	2010	animal_model	2	0
35	A chromosomally encoded virulence factor protects the Lyme disease pathogen against host-adaptive immunity.	2009	animal_model	2	0
37	Molecular adaptation of Borrelia burgdorferi in the murine host.	2002	animal_model	2	0
38	Coiling phagocytosis of Borrelia burgdorferi by primary human macrophages is controlled by CDC42Hs and Rac1 and involves recruitment of Wiskott-Ald...	2001	in_vitro	2	0
39	Inhibition of Fibroblast Apoptosis by *Borrelia afzelii*, *Coxiella burnetii* and *Bartonella henselae*	2011	in_vitro	1	1
40	Susceptibility of Borrelia afzelii strains to antimicrobial agents.	2005	in_vitro	1	1
41	Borrelia burgdorferi RST1 (OspC Type A) Genotype Is Associated with Greater Inflammation and More Severe Lyme Disease	2011	in_vitro	1	0
42	Interferon-gamma production in Lyme arthritis synovial tissue promotes differentiation of fibroblast-like synoviocytes into immune effector cells	2019	in_vitro	1	0
43	The FGF/FGFR system in the microglial neuroinflammation with Borrelia burgdorferi: likely intersectionality with other neurological conditions	2023	in_vitro	1	0

Continued on next page

Table S9: Influential cited preclinical targets excluded from the 24-paper extraction subset (continued)

Candidate list rank	Paper	Year	Design	Citing records	Guideline cites
44	Induction of Interleukin 10 by Borrelia burgdorferi Is Regulated by the Action of CD14-Dependent p38 Mitogen-Activated Protein Kinase and cAMP-Medi...	2018	animal_model	1	0
45	Genetic Background Amplifies the Effect of Immunodeficiency in Antibiotic Efficacy Against Borrelia burgdorferi	2021	animal_model	1	0
46	Evaluation of Disulfiram Drug Combinations and Identification of Other More Effective Combinations against Stationary Phase Borrelia burgdorferi.	2020	in_vitro	1	0
47	Effect of dapsone alone and in combination with intracellular antibiotics against the biofilm form of B. burgdorferi.	2020	in_vitro	1	0
48	Identification of Essential Oils with Strong Activity against Stationary Phase Borrelia burgdorferi.	2018	in_vitro	1	0
49	Antimicrobial Activity of Bee Venom and Melittin against Borrelia burgdorferi.	2017	in_vitro	1	0
50	Ceftriaxone Pulse Dosing Fails to Eradicate Biofilm-Like Microcolony B. burgdorferi Persisters Which Are Sterilized by Daptomycin/ Doxycycline/Cefu...	2016	in_vitro	1	0
51	Eradication of Biofilm-Like Microcolony Structures of Borrelia burgdorferi by Daunomycin and Daptomycin but not Mitomycin C in Combination with Dox...	2016	in_vitro	1	0
52	Identification of Additional Anti-Persister Activity against Borrelia burgdorferi from an FDA Drug Library.	2015	in_vitro	1	0
53	Identification of new compounds with high activity against stationary phase Borrelia burgdorferi from the NCI compound collection.	2015	in_vitro	1	0
54	Identification and characterization of the factor H and FHL-1 binding complement regulator-acquiring surface protein 1 of the Lyme disease spiroche...	2009	in_vitro	1	0
55	Microglia are mediators of Borrelia burgdorferi-induced apoptosis in SH-SY5Y neuronal cells.	2009	in_vitro	1	0
56	Populations of human T lymphocytes that traverse the vascular endothelium stimulated by Borrelia burgdorferi are enriched with cells that secrete g...	2004	in_vitro	1	0
57	Molecular Mimicry in Lyme Arthritis Demonstrated at the Single Cell Level: LFA-1αLIs a Partial Agonist for Outer Surface Protein A-Reactive T Cells	2001	in_vitro	1	0
58	Borrelia burgdorferi-Induced Tolerance as a Model of Persistence via Immunosuppression	2003	in_vitro	1	0
59	A Review of Antibiotic-Tolerant Persisters and Their Relevance to Posttreatment Lyme Disease Symptoms.	2020	in_vitro	1	0
60	The Functional and Molecular Effects of Doxycycline Treatment on *Borrelia burgdorferi* Phenotype	2019	in_vitro	1	0
61	Middle region of the Borrelia burgdorferi surface-located protein 1 (Lmp1) interacts with host chondroitin-6-sulfate and independently facilitates...	2016	animal_model	1	0
62	Role of interleukin-23 (IL-23) receptor signaling for IL-17 responses in human Lyme disease.	2011	in_vitro	1	0
63	Increased caspase activity primes human lyme arthritis synovial $\gamma\delta$ T cells for proliferation and death (160.11)	2011	in_vitro	1	0
64	HLA-DR alleles determine responsiveness to Borrelia burgdorferi antigens in a mouse model of self-perpetuating arthritis.	2009	animal_model	1	0
65	CD14 Signaling Restrains Chronic Inflammation through Induction of p38-MAPK/SOCS-Dependent Tolerance	2009	in_vitro	1	0
66	The absence of linear plasmid 25 or 28-1 of Borrelia burgdorferi dramatically alters the kinetics of experimental infection via distinct mechanisms.	2003	animal_model	1	0
67	Conversion of Borrelia garinii cystic forms to motile spirochetes in vivo.	2001	animal_model	1	0
69	Multiple cross-reactive self-ligands for Borrelia burgdorferi-specific HLA-DR4-restricted T cells.	2000	in_vitro	1	0
70	Microbial lipopeptides induce the production of IL-17 in Th cells.	2000	in_vitro	1	0

Note: Rank refers to the paper's position in the full citation-overlap candidate list before final selection of the 24-paper extraction subset. Non-consecutive ranks indicate that higher-ranked records were either selected into the extraction subset or excluded at earlier screening stages.

REFERENCES

Aberer, E. and Wutte, N. (2016). Atrophosclerodermic manifestations of lyme borreliosis. *The Open Dermatology Journal* 10. doi:10.2174/1874372201610010027

Adkison, H. and Embers, M. E. (2023). Lyme disease and the pursuit of a clinical cure. *Frontiers in Medicine* 10, 1183344. doi:10.3389/fmed.2023.1183344

Alitalo, A., Meri, T., Lankinen, H., Seppälä, I., Lahdenne, P., Hefty, P. S., et al. (2002). Complement inhibitor factor h binding to lyme disease spirochetes is mediated by inducible expression of multiple plasmid-encoded outer surface protein e paralogs. *The Journal of Immunology* 169, 3847–3853. doi:10.4049/jimmunol.169.7.3847

Aslam, B., Nisar, M. A., Khurshid, M., and Farooq Salamat, M. K. (2017). Immune escape strategies of borrelia burgdorferi. *Future Microbiology* 12, 1219–1237. doi:10.2217/fmb-2017-0013

Bai, N. A. and Richardson, C. S. (2023). Posttreatment lyme disease syndrome and myalgic encephalomyelitis/chronic fatigue syndrome: A systematic review and comparison of pathogenesis. *Chronic Diseases and Translational Medicine* 9, 183–190. doi:10.1002/cdt3.74

Bankhead, T. (2016). Role of the vlse lipoprotein in immune avoidance by the lyme disease spirochete borrelia burgdorferi. *Onco Therapeutics* 7. doi:10.1615/forumimmundisther.2017019625

Bashchobanov, D. H., Stamatova, E., Andonova, R., Dragusheva, E., Gadzhovska, V., Popov, G., et al. (2024). Lyme neuroborreliosis in the context of dementia syndromes. *Cureus* 16. doi:10.7759/cureus.67057

Behera, A. K., Hildebrand, E., Scagliotti, J., Steere, A. C., and Hu, L. T. (2005). Induction of host matrix metalloproteinases by borrelia burgdorferi differs in human and murine lyme arthritis. *Infection and immunity* 73, 126–134. doi:10.1128/iai.73.1.126-134.2005

Berende, A., ter Hofstede, H. J., Vos, F. J., van Middendorp, H., Vogelaar, M. L., Tromp, M., et al. (2016). Randomized trial of longer-term therapy for symptoms attributed to lyme disease. *New England Journal of Medicine* 374, 1209–1220

Bever, C., Gronseth, G., Krupp, L., Wormser, G., Dotevall, L., Belman, A., et al. (2007). Practice Parameter: Treatment of nervous system Lyme disease: An evidence-based review: Report of the quality standards subcommittee of the american academy of neurology. *Neurology* 69, 91–102. doi:10.1212/01.wnl.0000265517.66976.28

Bockenstedt, L. K. and Belperron, A. A. (2024). Insights from omics in lyme disease. *The Journal of Infectious Diseases* 230, S18–S26. doi:10.1093/infdis/jiae250

Bockenstedt, L. K., Gonzalez, D. G., Haberman, A. M., Belperron, A. A., et al. (2012). Spirochete antigens persist near cartilage after murine lyme borreliosis therapy. *The Journal of clinical investigation* 122, 2652–2660. doi:10.1172/jci58813

Bogdos, M., Giannopoulos, S., and Kosmidou, M. (2016). The conflict on posttreatment lyme disease syndrome: a clinical mini review. *Neuroimmunology and Neuroinflammation* 3, 10–13. doi:10.4103/2347-8659.171605

Borgermans, L., Goderis, G., Vandevoorde, J., and Devroey, D. (2014). Relevance of chronic lyme disease to family medicine as a complex multidimensional chronic disease construct: a systematic review. *International Journal of Family Medicine* 2014, 138016. doi:10.1155/2014/138016

Brouwer, M. A., van De Schoor, F. R., Vrijmoeth, H. D., Netea, M. G., and Joosten, L. A. (2020). A joint effort: The interplay between the innate and the adaptive immune system in lyme arthritis. *Immunological Reviews* 294, 63–79. doi:10.1111/imr.12837

Cameron, D. J., Johnson, L. B., and Maloney, E. L. (2014). Evidence assessments and guideline recommendations in lyme disease: the clinical management of known tick bites, erythema migrans rashes and persistent disease. *Expert review of anti-infective therapy* 12, 1103–1135

Chalada, M. J., Stenos, J., and Bradbury, R. S. (2016). Is there a lyme-like disease in australia? summary of the findings to date. *One Health* 2, 42–54. doi:10.1016/j.onehlt.2016.03.003

Chang, Y.-F., Novosel, V., Chang, C.-F., Summers, B. A., Ma, D.-P., Chiang, Y.-W., et al. (2001). Experimental induction of chronic borreliosis in adult dogs exposed to borrelia burgdorferi–infected ticks and treated with dexamethasone. *American journal of veterinary research* 62, 1104–1112. doi:10.2460/ajvr.2001.62.1104

Crossland, N. A., Alvarez, X., and Embers, M. E. (2018). Late disseminated lyme disease: associated pathology and spirochete persistence posttreatment in rhesus macaques. *The American journal of pathology* 188, 672–682. doi:10.1016/j.ajpath.2017.11.005

Custodio, M. M., Sparks, J., and Long, T. E. (2022). Disulfiram: A repurposed drug in preclinical and clinical development for the treatment of infectious diseases. *Anti-infective agents* 20, 34–45. doi:10.2174/2211352520666220104104747

Dattwyler, R. J., Wormser, G. P., Rush, T. J., Finkel, M. F., Schoen, R. T., Grunwaldt, E., et al. (2005). A comparison of two treatment regimens of ceftriaxone in late lyme disease. *Wiener Klinische Wochenschrift* 117, 393–397

Dersch, R. and Rauer, S. (2023). Efficacy and safety of pharmacological treatments for lyme neuroborreliosis: An updated systematic review. *European journal of neurology* 30, 3780–3788. doi:10.1111/ene.16034

Dersch, R., Torbahn, G., and Rauer, S. (2024). Treatment of post-treatment lyme disease symptoms—a systematic review. *European Journal of Neurology* 31, e16293. doi:10.1111/ene.16293

Dumler, J. S. (2001). Molecular diagnosis of lyme disease: review and meta-analysis. *Molecular Diagnosis* 6, 1–11. doi:10.1007/bf03262098

Elsner, R. A., Hastey, C. J., Olsen, K. J., and Baumgarth, N. (2015). Suppression of long-lived humoral immunity following borrelia burgdorferi infection. *PLoS pathogens* 11, e1004976. doi:10.1371/journal.ppat.1004976

Embers, M. E., Barthold, S. W., Borda, J. T., Bowers, L., Doyle, L., Hodzic, E., et al. (2012). Persistence of borrelia burgdorferi in rhesus macaques following antibiotic treatment of disseminated infection. *PloS one* 7, e29914. doi:10.1371/journal.pone.0029914

Embers, M. E., Ramamoorthy, R., and Philipp, M. T. (2004). Survival strategies of borrelia burgdorferi, the etiologic agent of lyme disease. *Microbes and Infection* 6, 312–318. doi:10.1016/j.micinf.2003.11.014

Fallon, B. A., Keilp, J. G., Corbera, K. M., Petkova, E., Britton, C. B., Dwyer, E., et al. (2008). A randomized, placebo-controlled trial of repeated iv antibiotic therapy for lyme encephalopathy. *Neurology* 70, 992–1003

Feng, J., Auwaerter, P. G., and Zhang, Y. (2015). Drug combinations against borrelia burgdorferi persisters in vitro: eradication achieved by using daptomycin, cefoperazone and doxycycline. *PloS one* 10, e0117207. doi:10.1371/journal.pone.0117207

Feng, J., Shi, W., Zhang, S., Sullivan, D., Auwaerter, P. G., and Zhang, Y. (2016). A drug combination screen identifies drugs active against amoxicillin-induced round bodies of in vitro borrelia burgdorferi persisters from an fda drug library. *Frontiers in microbiology* 7, 743. doi:10.3389/fmicb.2016.00743

Feng, J., Wang, T., Shi, W., Zhang, S., Sullivan, D., Auwaerter, P. G., et al. (2014). Identification of novel activity against borrelia burgdorferi persisters using an fda approved drug library. *Emerging microbes & infections* 3, 1–8. doi:10.1038/emi.2014.53

Gaubitz, M., Dressler, F., Huppertz, H., Krause, A., and die Kommission Pharmakotherapie der DGRh (2014). Diagnostik und therapie der lyme-arthritis. *Zeitschrift für Rheumatologie* 73, 469–474. doi:10.1007/s00393-014-1370-7

Goc, A. and Rath, M. (2016). The anti-borreliae efficacy of phytochemicals and micronutrients: an update. *Therapeutic advances in infectious disease* 3, 75–82. doi:10.1177/2049936116655502

Guérin, M., Shawky, M., Zedan, A., Octave, S., Avalle, B., Maffucci, I., et al. (2023). Lyme borreliosis diagnosis: state of the art of improvements and innovations. *BMC microbiology* 23, 204. doi:10.1186/s12866-023-02935-5

Gueye, S., Seck, S. M., Kane, Y., Tosi, P.-O., Dahri, S., Kounde, C., et al. (2019). La néphrite de lyme chez l'homme: bases physiopathologiques et spectre lésionnel rénal. *Néphrologie & Thérapeutique* 15, 127–135. doi:10.1016/j.nephro.2018.09.004

Hartiala, P., Hytönen, J., Pelkonen, J., Kimppa, K., West, A., Penttinen, M. A., et al. (2007). Transcriptional response of human dendritic cells to borrelia garinii: defective cd38 and ccr7 expression detected. *Journal of Leukocyte Biology* 82, 33–43. doi:10.1189/jlb.1106709

Hernandez-Ruiz, V., Letenneur, L., Fülöp, T., Helmer, C., Roubaud-Baudron, C., Avila-Funes, J.-A., et al. (2022). Infectious diseases and cognition: do we have to worry? *Neurological Sciences* 43, 6215–6224. doi:10.1007/s10072-022-06280-9

Hodzic, E., Feng, S., Holden, K., Freet, K. J., and Barthold, S. W. (2008). Persistence of borrelia burgdorferi following antibiotic treatment in mice. *Antimicrobial agents and chemotherapy* 52, 1728–1736. doi:10.1128/aac.01050-07

Hyde, J. A. (2017). Borrelia burgdorferi keeps moving and carries on: a review of borrelial dissemination and invasion. *Frontiers in immunology* 8, 114. doi:10.3389/fimmu.2017.00114

Iyer, R., Mukherjee, P., Wang, K., Simons, J., Wormser, G. P., and Schwartz, I. (2013). Detection of borrelia burgdorferi nucleic acids after antibiotic treatment does not confirm viability. *Journal of clinical microbiology* 51, 857–862. doi:10.1128/jcm.02785-12

Jakubowska, K., Janocha, A., Jerzak, A., and Ziemba, P. (2024). Diagnosis, clinical manifestations and treatment of lyme disease. *Quality in Sport* 18, 53283–53283. doi:10.12775/qs.2024.18.53283

Jarosz, A. C. and Badawi, A. (2019). Metabolites of prostaglandin synthases as potential biomarkers of lyme disease severity and symptom resolution. *Inflammation Research* 68, 7–17. doi:10.1007/s00011-018-1180-5

Jaulhac, B., Saunier, A., Caumes, E., Bouiller, K., Gehanno, J., Rabaud, C., et al. (2019). Lyme borreliosis and other tick-borne diseases. guidelines from the french scientific societies (ii). biological diagnosis, treatment, persistent symptoms after documented or suspected lyme borreliosis. *Médecine et Maladies Infectieuses* 49, 335–346. doi:10.1016/j.medmal.2019.05.001

Jutras, B. L., Lochhead, R. B., Kloos, Z. A., Biboy, J., Strle, K., Booth, C. J., et al. (2019). Borrelia burgdorferi peptidoglycan is a persistent antigen in patients with lyme arthritis. *Proceedings of the National Academy of Sciences* 116, 13498–13507. doi:10.1073/pnas.1904170116

Klempner, M. S., Hu, L. T., Evans, J., Schmid, C. H., Johnson, G. M., Trevino, R. P., et al. (2001). Two controlled trials of antibiotic treatment in patients with persistent symptoms and a history of lyme disease. *New England Journal of Medicine* 345, 85–92. doi:10.1056/NEJM200107123450202

Kraiczy, P. (2016). Hide and seek: how lyme disease spirochetes overcome complement attack. *Frontiers in immunology* 7, 385. doi:10.3389/fimmu.2016.00385

Kraiczy, P., Hellwage, J., Skerka, C., Becker, H., Kirschfink, M., Simon, M. M., et al. (2004). Complement resistance of borrelia burgdorferi correlates with the expression of bbcrasp-1, a novel linear plasmid-encoded surface protein that interacts with human factor h and fhl-1 and is unrelated to erp proteins. *Journal of Biological Chemistry* 279, 2421–2429. doi:10.1074/jbc.m308343200

Krupp, L., Hyman, L., Grimson, R., Coyle, P., Melville, P., Ahnn, S., et al. (2003). Study and treatment of post lyme disease (stop-ld) a randomized double masked clinical trial. *Neurology* 60, 1923–1930

Liang, F. T., Brown, E. L., Wang, T., Iozzo, R. V., and Fikrig, E. (2004). Protective niche for borrelia burgdorferi to evade humoral immunity. *The American journal of pathology* 165, 977–985. doi:10.1016/s0002-9440(10)63359-7

Liang, F. T., Jacobs, M. B., Bowers, L. C., and Philipp, M. T. (2002). An immune evasion mechanism for spirochetal persistence in lyme borreliosis. *The Journal of experimental medicine* 195, 415–422. doi:10.1084/jem.20011870

Lin, Y.-P., Li, L., Zhang, F., and Linhardt, R. J. (2017). Borrelia burgdorferi glycosaminoglycan-binding proteins: a potential target for new therapeutics against lyme disease. *Microbiology* 163, 1759–1766. doi:10.1099/mic.0.000571

Livengood, J. A. and Gilmore Jr, R. D. (2006). Invasion of human neuronal and glial cells by an infectious strain of borrelia burgdorferi. *Microbes and infection* 8, 2832–2840. doi:10.1016/j.micinf.2006.08.014

Miklossy, J. (2011). Alzheimer's disease-a neurospirochetosis. analysis of the evidence following koch's and hill's criteria. *Journal of neuroinflammation* 8, 90. doi:10.1186/1742-2094-8-90

Miklossy, J. (2012). Chronic or late lyme neuroborreliosis: analysis of evidence compared to chronic or late neurosyphilis. *The open neurology journal* 6, 146. doi:10.2174/1874205x01206010146

Miklossy, J., Kasas, S., Zurn, A. D., McCall, S., Yu, S., and McGeer, P. L. (2008). Persisting atypical and cystic forms of borrelia burgdorferi and local inflammation in lyme neuroborreliosis. *Journal of neuroinflammation* 5, 40. doi:10.1186/1742-2094-5-40

Motamed, M., Liblik, K., Miranda-Arboleda, A. F., Wamboldt, R., Wang, C. N., Cingolani, O., et al. (2023). Disseminated lyme disease and dilated cardiomyopathy: a systematic review. *Trends in Cardiovascular Medicine* 33, 531–536. doi:10.1016/j.tcm.2022.05.010

Mygland, Å., Ljøstad, U., Fingerle, V., Rupprecht, T., Schmutzhard, E., and Steiner, I. (2010). Efns guidelines on the diagnosis and management of european lyme neuroborreliosis. *European journal of neurology* 17, 8–e4. doi:10.1111/j.1468-1331.2009.02862.x

Oksi, J., Nikoskelainen, J., Hiekkanen, H., Lauhio, A., Peltomaa, M., Pitkäranta, A., et al. (2007). Duration of antibiotic treatment in disseminated lyme borreliosis: a double-blind, randomized, placebo-controlled, multicenter clinical study. *European Journal of Clinical Microbiology & Infectious Diseases* 26, 571–581. doi:10.1007/s10096-007-0340-2

Pothineni, V. R., Potula, H.-H. S., Ambati, A., Mallajosyula, V. V. A., Sridharan, B., Inayathullah, M., et al. (2020). Azlocillin can be the potential drug candidate against drug-tolerant borrelia burgdorferi sensu stricto jlb31. *Scientific reports* 10, 3798. doi:10.1038/s41598-020-59600-4

Raffetin, A., Saunier, A., Bouiller, K., Caraux-Paz, P., Eldin, C., Gallien, S., et al. (2020). Unconventional diagnostic tests for lyme borreliosis: a systematic review. *Clinical Microbiology and Infection* 26, 51–59. doi:10.1016/j.cmi.2019.06.033

Rauer, S., Kastenbauer, S., Fingerle, V., Hunfeld, K.-P., Huppertz, H.-I., Dersch, R., et al. (2018). Lyme neuroborreliosis. *Deutsches Ärzteblatt International* 115, 751. doi:10.3238/arztebl.2018.0751

Rebman, A. W. and Aucott, J. N. (2020). Post-treatment lyme disease as a model for persistent symptoms in lyme disease. *Frontiers in medicine* 7, 524338. doi:10.3389/fmed.2020.00057

Sapi, E., Kaur, N., Anyanwu, S., Luecke, D. F., Datar, A., Patel, S., et al. (2011). Evaluation of in-vitro antibiotic susceptibility of different morphological forms of borrelia burgdorferi. *Infection and drug resistance* , 97–113doi:10.2147/idr.s19201

Shapiro, E. D. (2014). Borrelia burgdorferi (lyme disease). *Pediatrics in review* 35, 500–509. doi:10.1542/pir.35-12-500

Sharma, B., Brown, A. V., Matluck, N. E., Hu, L. T., and Lewis, K. (2015). Borrelia burgdorferi, the causative agent of lyme disease, forms drug-tolerant persister cells. *Antimicrobial agents and chemotherapy* 59, 4616–4624. doi:10.1128/aac.00864-15

Shor, S., Green, C., Szantyr, B., Phillips, S., Liegner, K., Burrascano Jr, J., et al. (2019). Chronic lyme disease: an evidence-based definition by the ilads working group. *Antibiotics* 8, 269. doi:10.3390/antibiotics8040269

Skripchenko, E. Y., Ivanova, G., Skripchenko, N., and Egorova, E. (2022). Modern concepts on the pathogenesis of neuroborreliosis. *Zhurnal Nevrologii i Psikhiatrii imeni SS Korsakova* 122, 27–35. doi:10.17116/jnevro202212207127

Stinco, G., Bergamo, S., et al. (2016). Impact of co-infections in lyme disease. *The Open Dermatology Journal* 10, 55–61. doi:10.2174/1874372201610010055

Strzoda, A., Kamińska, M., Strzoda, A., Juda, A., Strzoda, A., and Sowiński, W. (2023). Borrelia burgdorferi sensu lato infection-induced autoimmunity–a decennary literature review doi:10.2478/jhsm-2023-0004

Talbot, N. C., Spillers, N. J., Luther, P., Flanagan, C., Soileau, L. G., Ahmadzadeh, S., et al. (2023). Lyme disease and post-treatment lyme disease syndrome: current and developing treatment options. *Cureus* 15

Thompson, D., Watt, J. A., and Brissette, C. A. (2021). Host transcriptome response to borrelia burgdorferi sensu lato. *Ticks and tick-borne diseases* 12, 101638. doi:10.1016/j.ttbdis.2020.101638

Tracy, K. E. and Baumgarth, N. (2017). Borrelia burgdorferi manipulates innate and adaptive immunity to establish persistence in rodent reservoir hosts. *Frontiers in immunology* 8, 116. doi:10.3389/fimmu.2017.00116

Verhaegh, D., Joosten, L. A., and Oosting, M. (2017). The role of host immune cells and borrelia burgdorferi antigens in the etiology of lyme disease. *European cytokine network* 28, 70–84. doi:10.1684/ecn.2017.0396

Verschoor, Y., Vrijlandt, A., Spijker, R., Van Hest, R., Ter Hofstede, H., van Kempen, K., et al. (2022). Persistent borrelia burgdorferi sensu lato infection after antibiotic treatment: Systematic overview and appraisal of the current evidence from experimental animal models. *Clinical Microbiology Reviews* 35, e00074–22. doi:10.1128/cmr.00074-22

Woitzik, P. and Linder, S. (2021). Molecular mechanisms of borrelia burgdorferi phagocytosis and intracellular processing by human macrophages. *Biology* 10, 567. doi:10.3390/biology10070567

Wong, K. H., Shapiro, E. D., and Soffer, G. K. (2022). A review of post-treatment lyme disease syndrome and chronic lyme disease for the practicing immunologist. *Clinical reviews in allergy & immunology* 62, 264–271. doi:10.1007/s12016-021-08906-w

Wormser, G. P., Dattwyler, R. J., Shapiro, E. D., Halperin, J. J., Steere, A. C., Klempner, M. S., et al. (2006). The clinical assessment, treatment, and prevention of lyme disease, human granulocytic anaplasmosis, and babesiosis: clinical practice guidelines by the infectious diseases society of america. *Clinical Infectious Diseases* 43, 1089–1134. doi:10.1086/508667

Yrjänaäinen, H., Hytönen, J., Song, X.-y. R., Oksi, J., Hartiala, K., and Viljanen, M. K. (2007). Anti—tumor necrosis factor—α treatment activates borrelia burgdorferi spirochetes 4 weeks after ceftriaxone treatment in c3h/he mice. *The Journal of infectious diseases* 195, 1489–1496. doi:10.1086/513873

Yrjänäinen, H., Hytönen, J., Hartiala, P., Oksi, J., and Viljanen, M. K. (2010). Persistence of borrelial dna in the joints of borrelia burgdorferi-infected mice after ceftriaxone treatment. *Apmis* 118, 665–673. doi:10.1111/j.1600-0463.2010.02615.x

Zadeh, M. M. (2007). Traitement et suivi des phases secondaire et tertiaire de la borréliose de lyme. *Médecine et maladies infectieuses* 37, 368–380. doi:10.1016/j.medmal.2006.01.030

Zhang, X., Jiang, Y., Chen, Y., Yang, J., Zhang, X., Xing, L., et al. (2023). Efficacy and safety of antibiotic therapy for post-lyme disease? a systematic review and network meta-analysis. *BMC Infectious Diseases* 23, 22. doi:10.1186/s12879-023-07989-4

Zimmerli, S. (2016). Update of the swiss guidelines on post-treatment lyme disease syndrome. *Swiss medical weekly* doi:10.4414/smw.2016.14353

Zubair Alam, M., Alam, Q., Amjad Kamal, M., Ahmad Jiman-Fatani, A., I Azhar, E., Azhar Khan, M., et al. (2017). Infectious agents and neurodegenerative diseases: exploring the links. *Current topics in medicinal chemistry* 17, 1390–1399. doi:10.2174/1568026617666170103164040
